# From medical necessity to private-sector dominance: a two-decade evolution of caesarean section determinants in Bangladesh (2004–2022)

**DOI:** 10.1038/s41598-026-52229-9

**Published:** 2026-05-09

**Authors:** Ashiqur Rahman Rony, Arman Hossen, Abida Sultana Asha, Kazi Sabbir Ahmad Nahin

**Affiliations:** 1 Department of Statistics, Government Titumir College, Dhaka, Bangladesh; 2 Data Analytics, Development Research Initiative (dRi), Dhaka, Bangladesh; 3 Department of Statistics, Miami University, Oxford, OH USA; 4 Department of Statistics, College of Arts and Sciences, University of Kentucky, Lexington, KY USA; 5 Department of Quantitative Sciences, International University of Business Agriculture and Technology, Dhaka, Bangladesh

**Keywords:** Caesarean section, Bangladesh, Trend, Private sector, Socioeconomic inequality, Antenatal care, Regional variation, Survey-weighted multivariable logistic regression, BDHS, Health care, Medical research, Risk factors

## Abstract

Caesarean section (CS) is a critical life-saving intervention when medically directed, yet its prevalence in Bangladesh has surged impulsively from approximately 4% in 2004 to nearly 45% in 2022, highlighting a distinctive South Asian pattern of widespread surgical overuse. This study examines the shifting socioeconomic and geographic determinants of CS, specifically investigating the private sector’s role in widening inequalities and the transition of surgical birth from a targeted medical necessity to a routine practice dominated by private-sector utilization. We analyzed Bangladesh Demographic and Health Survey (BDHS) data of 26,845 ever-married women, covering the periods 2004–2007, 2011–2014, and 2018–2022. Variables were harmonized and administrative divisions grouped into broader regions to ensure comparability. Using survey-weighted multivariable logistic regression, we identified independent predictors of caesarean section, adjusting for sociodemographic, clinical, and geographic factors while accounting for complex survey design. Weighted CS prevalence increased sharply, rising from 6.8% in 2004–2007 to 39% in 2018–2022. This surge was largely driven by the private sector, where CS rates expanded from roughly 55% in the earliest period to 84% in the most recent. Longitudinal analyses also revealed a notable shift in key determinants: medical indications, initially strong predictors in 2004–2007 (e.g., Multiple Births AOR 9.55), had substantially weaker effects by 2018–2022 (AOR 2.93), indicating growing widespread saturation of CS beyond clinical necessity. The influence of frequent antenatal care similarly declined over time (AOR 9.62 to 4.62), though it remained a significant predictor. Socioeconomic patterns shifted as well; women in the “poorer” wealth quintile moved from having lower odds of CS in 2004–2007 (AOR 0.53) to higher odds in 2018–2022 (AOR 1.45), suggesting increased penetration of surgical delivery into lower-income groups. Geographically, residence in Coastal areas consistently remained protective relative to the Central region. Caesarean delivery in Bangladesh has shifted from a life-saving procedure to a highly prevalent practice, driven predominantly by the expansion of private-sector facilities. An unregulated private sector, rising healthcare contact, and socioeconomic disparities now fuel overuse, demanding stronger regulation, clinical audits, and expanded midwifery‑led care to ensure appropriate use.

**Supplementary Information:**

The online version contains supplementary material available at 10.1038/s41598-026-52229-9.

## Introduction

Caesarean section (CS) is a common obstetric procedure used to deliver a baby through a surgical incision when clinically indicated. Although this procedure can save maternal and neonatal lives when medically necessary, its overuse presents serious public health concerns, including obstructed labor, fetal distress, abnormal fetal presentation, hypertensive disorders, placenta previa and future obstetric complications^1,2^. Additionally, the long-term immune effects on children born through CS, such as a higher risk of asthma and obesity, have led experts to call for a worldwide review of birthing practices^3,4^. Moreover, it not only creates complications for the mother and child but also places a financial burden on the family and the healthcare system. In 2018, approximately USD 483 million was paid in out-of-pocket expenses due to unnecessary CS deliveries^5,6^.

The global CS rate has risen rapidly over the past two decades, increasing from about 7% to around 21%, with substantial variation across countries and growing inequalities in access. The World Health Organization (WHO) emphasizes that CS can be lifesaving when medically indicated but does not recommend a specific CS rate for countries to achieve at the population level. Instead, WHO notes that, at the population level, increases in CS rates are associated with reductions in maternal and newborn mortality up to about 10%, while rates above this level are not associated with further mortality reductions^3,7^. In lower- and middle-income countries (LMICs), particularly in parts of Asia, CS rates have increased sharply, often within dual health systems comprising under-resourced public sectors and rapidly expanding private sectors where financial incentives may favor surgical births^8^. According to WHO’s projections, global CS rate will reach nearly 30% by 2030^7^. In south Asia, particularly, Bangladesh, Nepal, and Pakistan have grown at alarming rates due to urbanization, maternal demand, and mostly outpacing improvements in obstetric care and quality and equity^9^. This “South Asian anomaly” is frequently attributed to the fast expansion of the private healthcare sector, which is characterized by a lack of strong regulatory control and financial models that favor surgical versus vaginal deliveries^10^.

According to BDHS (Bangladesh Demographic and Health Survey), the rate of CS was nearly 4% in 2004, and this rate has increased nearly ten times and reached approximately 46% in 2022; however, the pooled estimate for 2018–2022 was 39%, reflecting an average across multiple survey years within that period^11,12^. This rise suggests a transformation in CS use, from its original purpose as a life-saving intervention to a frequently performed, routine procedure that is now widely adopted across the healthcare system^12^.

However, this trend varies significantly based on geography and socioeconomic status. While CS rates in rural areas remain around 10%, they exceed 55% in urban centers^10,11^. These disparities indicate that factors such as access to private healthcare, higher maternal education, and household wealth often influence the mode of delivery more than clinical necessity^10,11,13^. Highly educated women often view CS as a modern and painless method of childbirth, frequently requesting the procedure even without medical indication^14^. Advanced maternal age (35+) is consistently associated with higher CS rates due to increased risks of complications such as gestational diabetes and hypertension^15^. The rising prevalence of maternal overweight and obesity in Bangladesh has also been identified as a significant biological factor driving surgical interventions^16,17^. Furthermore, primiparous women (first-time mothers) are more likely to undergo CS, often establishing a precedent of “once a caesarean, always a caesarean " in subsequent pregnancies^18,19^.

Based on previous research, maternal age, parity, and antenatal care (ANC) visits are significant factors influencing CS rates in Bangladesh^9,15,18,20–24^. However, the ANC Paradox, where increased utilization of private sector ANC correlates with elective CS rather than natural birth, requires further investigation^11^. In urban Bangladesh, the social perception of childbirth has shifted, with fear of labor pain and the scheduling convenience for families and physicians contributing to the decline of midwifery-led care^24,25^. To guide effective policy interventions, research should empirically examine provider-induced demand and the growing cultural acceptance of CS as a modern delivery method^14,25^. Although professional midwives have been introduced in the public sector, the private sector, which handles most urban deliveries, primarily focuses on surgical interventions. Consequently, a synthesis of existing data is essential to determine how socioeconomic advantages and systemic healthcare shortcomings contribute to elevated CS rates^26^.

While prior analyses have documented the sharp rise in CS rates from single-digit levels in the early 2000s to nearly half of all births by 2022, most have relied on single survey waves or limited time windows. This fragmented approach often fails to capture how the sociodemographic and systemic drivers of this increase have evolved over the long term. Consequently, a gap remains in understanding how the interplay between rising socioeconomic advantages and the unregulated expansion of private-sector facilities has reshaped the landscape of maternal healthcare delivery across the country.

In contrast to existing assessments, this study utilizes a harmonized, multi-wave analysis of nationally representative data spanning nearly two decades (2004–2022). By applying an appropriate analysis scheme, we seek to answer three critical questions: How have the socioeconomic and geographic determinants of CS delivery evolved over the past twenty years? To what extent has the private sector contributed to the widening inequality in surgical birth rates? And finally, are current trends shifting CS from a life-saving intervention to a routine private-sector practice among specific population groups? This longitudinal approach provides robust, nationally representative evidence which is necessary to disentangle the complex factors driving the surge in surgical deliveries.

The primary objective of this study is to evaluate how socioeconomic, maternal, and geographic determinants of CS have evolved over the past two decades. The secondary objective is to quantify the extent to which the private healthcare sector has driven the widening inequality in surgical birth rates. The insights generated by this analysis are intended to directly inform policy frameworks essential for meeting the Sustainable Development Goals (SDG), specifically SDG 3.1 (reducing global maternal mortality) and SDG 3.7 (ensuring universal access to sexual and reproductive health-care services). By identifying the specific drivers of inequality and provider-induced demand, our findings support the development of targeted interventions, such as strengthening clinical audit systems and dissociating financial incentives from surgical decisions, to ensure that obstetric care in Bangladesh remains both equitable and medically appropriate.

## Methodology

### Data source and study design

This study used data from the Bangladesh Demographic and Health Surveys (BDHS), nationally representative cross-sectional household surveys that periodically monitor health and population indicators^27^. The BDHS uses a stratified, two-stage cluster sampling design to select households and women aged 15–49 years for interview. BDHS sampling procedures, questionnaires, and fieldwork protocols are broadly comparable across survey rounds and have been described elsewhere. To assess long-term trends in caesarean section (CS) delivery and its determinants, we pooled BDHS survey waves into three sequential periods (2004–2007, 2011–2014, and 2018–2022) to increase statistical power. To ensure comparability across waves, variables were harmonized using core questionnaire items consistently available across rounds and were coded with uniform category definitions (e.g., maternal education and household wealth quintile). Because administrative boundaries can vary across survey years, we additionally grouped administrative divisions into broader geographic regions and used this harmonized residential region variable in pooled analyses.

All analyses accounted for the BDHS complex survey design (clustering, stratification, and sampling weights) to produce nationally representative estimates. For pooled analyses within each period, we re-scaled the sampling weights using population totals so that each survey round contributed in proportion to the population size at the time of data collection, thereby avoiding undue influence of any single survey wave.

### Analytic sample

Our study sample comprised ever-married women aged 15–49 years with a live birth in the three years preceding the survey and complete records on the mode of delivery. Women with missing or inconsistent information on key variables, including delivery mode, were excluded. Analyses were conducted using a complete-case approach after excluding observations with missing data on the outcome or included covariates. To minimize recall bias and ensure comparability across survey waves, only the most recent birth reported by each woman was included. Multiple births were retained in the sample, and multiple birth status was included as a covariate in multivariable analyses.

### Outcome variable

The primary outcome was caesarean section (CS) delivery for the most recent live birth. In the DHS, women were asked whether their most recent birth was delivered by caesarean section (delivery through a surgical incision in the abdomen and uterus)^28^. Responses were coded as a binary variable: 1 for caesarean delivery and 0 for non-caesarean (vaginal) delivery.

### Explanatory variables

Explanatory variables were selected based on prior literature on determinants of caesarean section delivery and on their consistent availability across all BDHS waves included in this study. These variables capture pregnancy- and birth-related characteristics, maternal use of healthcare services, sociodemographic conditions, and geographic context. To ensure comparability across survey periods, variables were coded following the BDHS recode manuals.

Pregnancy- and birth-related characteristics included place of delivery (home, public sector facility, private sector facility, NGO facility, or other), multiple birth status, sex of the child, and birth order (first, second, or third or higher). These factors reflect both childbirth circumstances and contextual conditions that may influence delivery practices. Place of delivery distinguishes institutional and non-institutional births and captures differences in service provision across facility types.

Maternal interaction with the healthcare system during pregnancy was measured using the number of antenatal care (ANC) visits, categorized as none, one to three, or four or more. This categorization aligns with DHS reporting and allows assessment of whether increasing contact with maternity care services is associated with caesarean delivery.

Sociodemographic variables included maternal age at birth (< 20, 20–24, 25–29, 30–34, 35–39, and ≥ 40 years), maternal body mass index (underweight, normal weight, overweight, or obese), and maternal and partner’s education levels (none, primary, secondary, or higher). Household socioeconomic status was measured using the DHS wealth quintile (poorest to richest). Maternal working status, media exposure, and religion were included to account for differences in social position, access to information, and cultural context that may influence healthcare-seeking behavior and delivery preferences.

Geographic factors included place of residence (urban or rural) and residential region (central, coastal, north, or south-west/east), capturing spatial variation in access to obstetric services and regional differences in health system context.

All explanatory variables were included in survey-weighted multivariable regression models to estimate their independent associations with caesarean delivery across pooled survey periods.

### Statistical analysis

Stata version 17 was used for data management and cleaning, including merging survey datasets, defining variables, and checking consistency across survey waves. Statistical analyses were conducted using R (version 4.4 or later).

Descriptive statistics were used to summarize participant characteristics across pooled survey periods. Chi-square tests were used to assess unadjusted associations between caesarean delivery and explanatory variables within each pooled period.

Survey-weighted multivariable logistic regression models were fitted separately for each pooled period (2004–2007, 2011–2014, and 2018–2022) to identify factors independently associated with caesarean delivery. All explanatory variables were included simultaneously to control for potential confounding. Results are reported as adjusted odds ratios (AORs) with 95% confidence intervals (CIs).

All analyses accounted for the BDHS complex survey design (stratification, clustering, and sampling weights) using appropriate survey design specifications. Specifically, sampling weights were normalized as recommended for DHS analyses, and the survey design was specified using the standard BDHS sampling structure (strata and cluster/primary sampling unit), so that all estimates and standard errors appropriately reflected the complex survey design. Multicollinearity was assessed using generalized variance inflation factors (GVIFs), and no evidence of problematic multicollinearity was observed. Statistical significance was defined as p-value < 0.05.

Temporal trends in caesarean prevalence were assessed by comparing weighted estimates across pooled survey periods. Trend figures were generated to visualize changes in caesarean delivery over time and across selected subgroups, and R was used for visualization and figure preparation.

Finally, to address potential confounding from home births and to isolate the determinants of surgical delivery among those accessing care, we conducted a sensitivity analysis restricting the multivariable logistic regression models exclusively to institutional deliveries. Survey-weighted adjusted odds ratios from this restricted sample were compared against the total population models to assess the robustness of socioeconomic and clinical predictors.

### Ethical considerations

This study used publicly available, anonymized secondary data obtained from the DHS Program with formal permission. Ethical approval for the original BDHS surveys was obtained by the relevant national and international institutional review boards, and informed consent was obtained from all participants during the original data collection. No additional ethical approval was required for this secondary analysis.

## Results

**Fig. 1:**
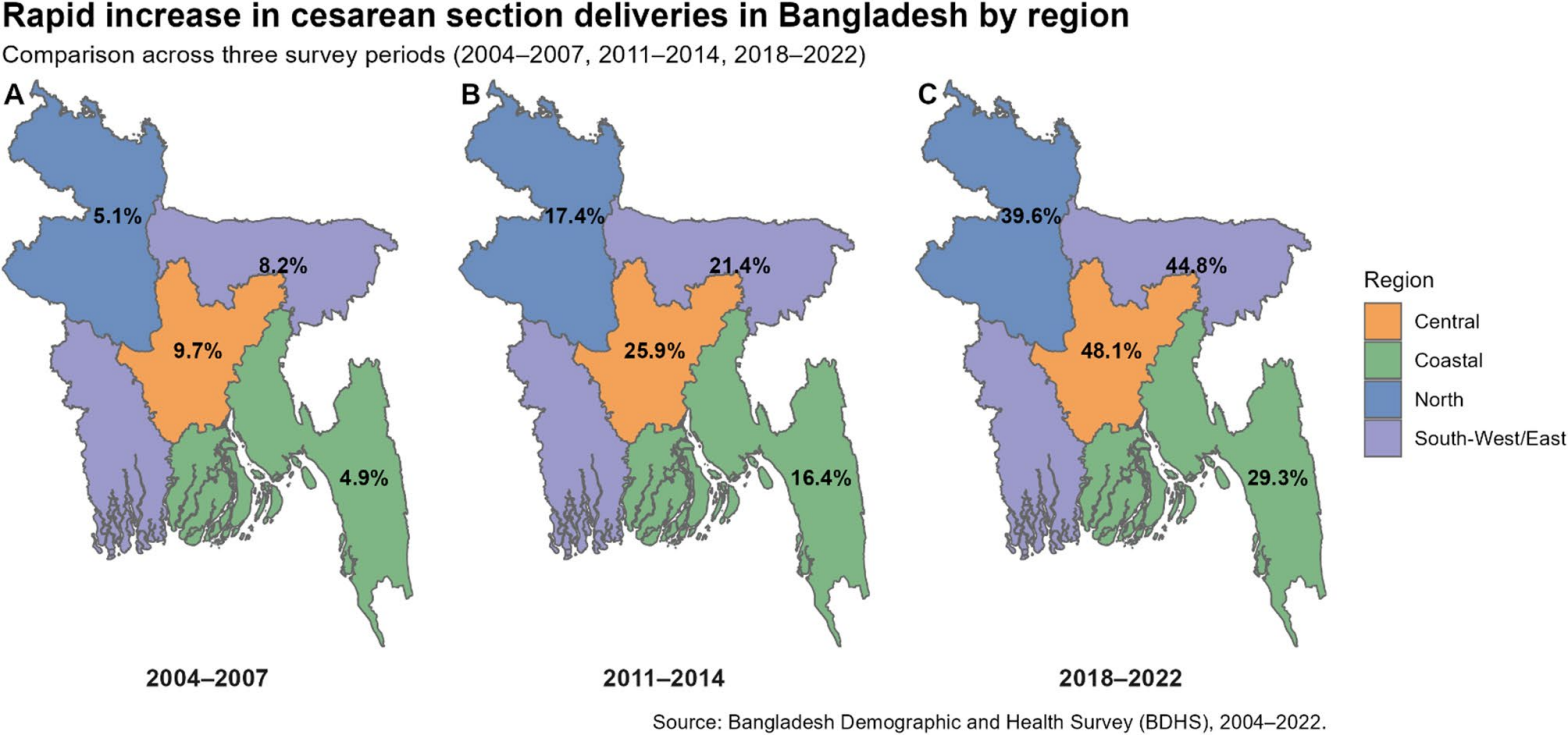
Geographical trends in caesarean section delivery prevalence across pooled Bangladesh Demographic and Health Survey periods, 2004–2022.

**Fig. 2:**
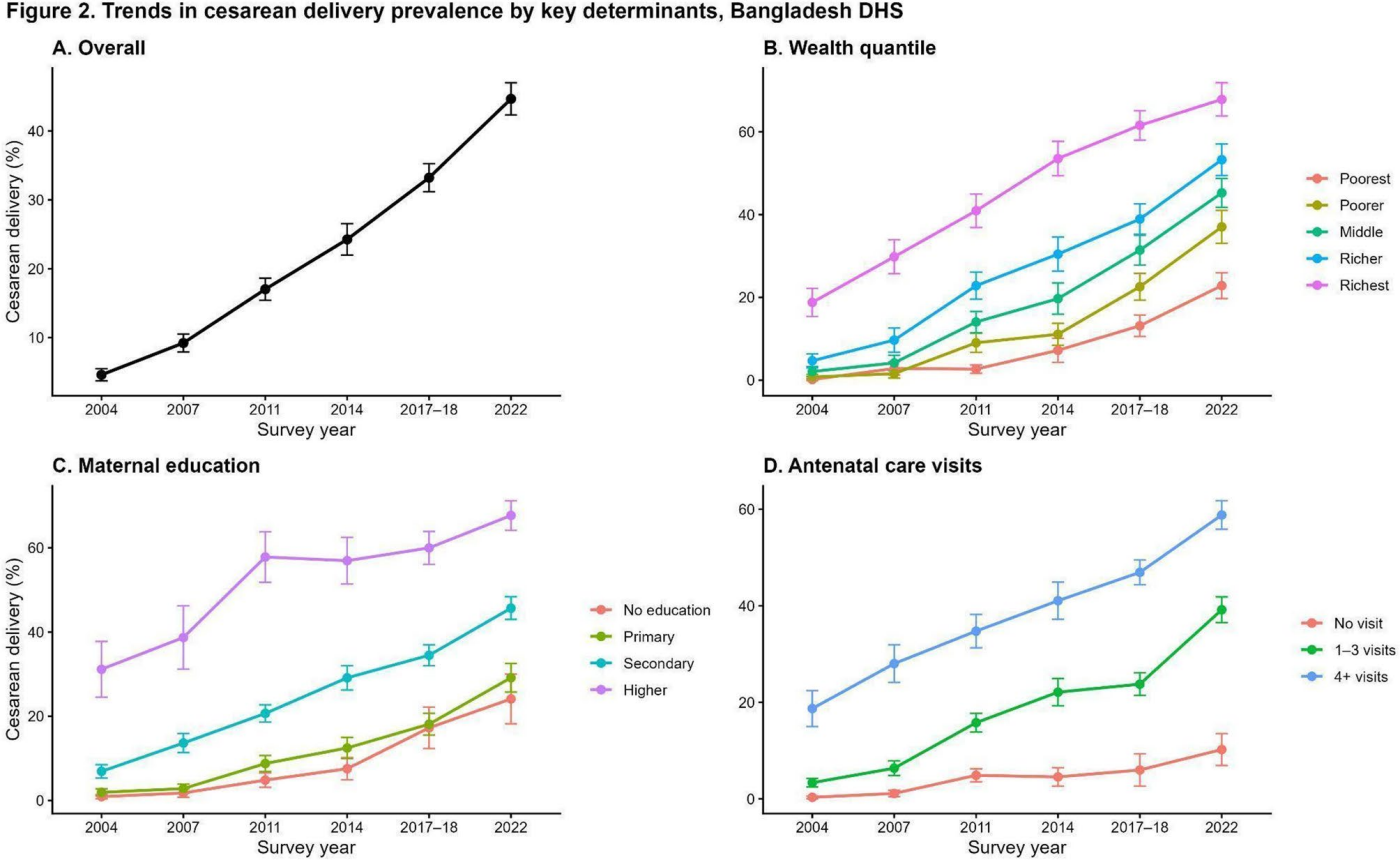
Trends in caesarean delivery prevalence by key determinants, Bangladesh DHS 2004–2022. (**A**) Overall; (**B**) household wealth quintile; (**C**) maternal education; (**D**) antenatal care visits. Points represent prevalence estimates by survey year with 95% confidence intervals.

**Fig. 3:**
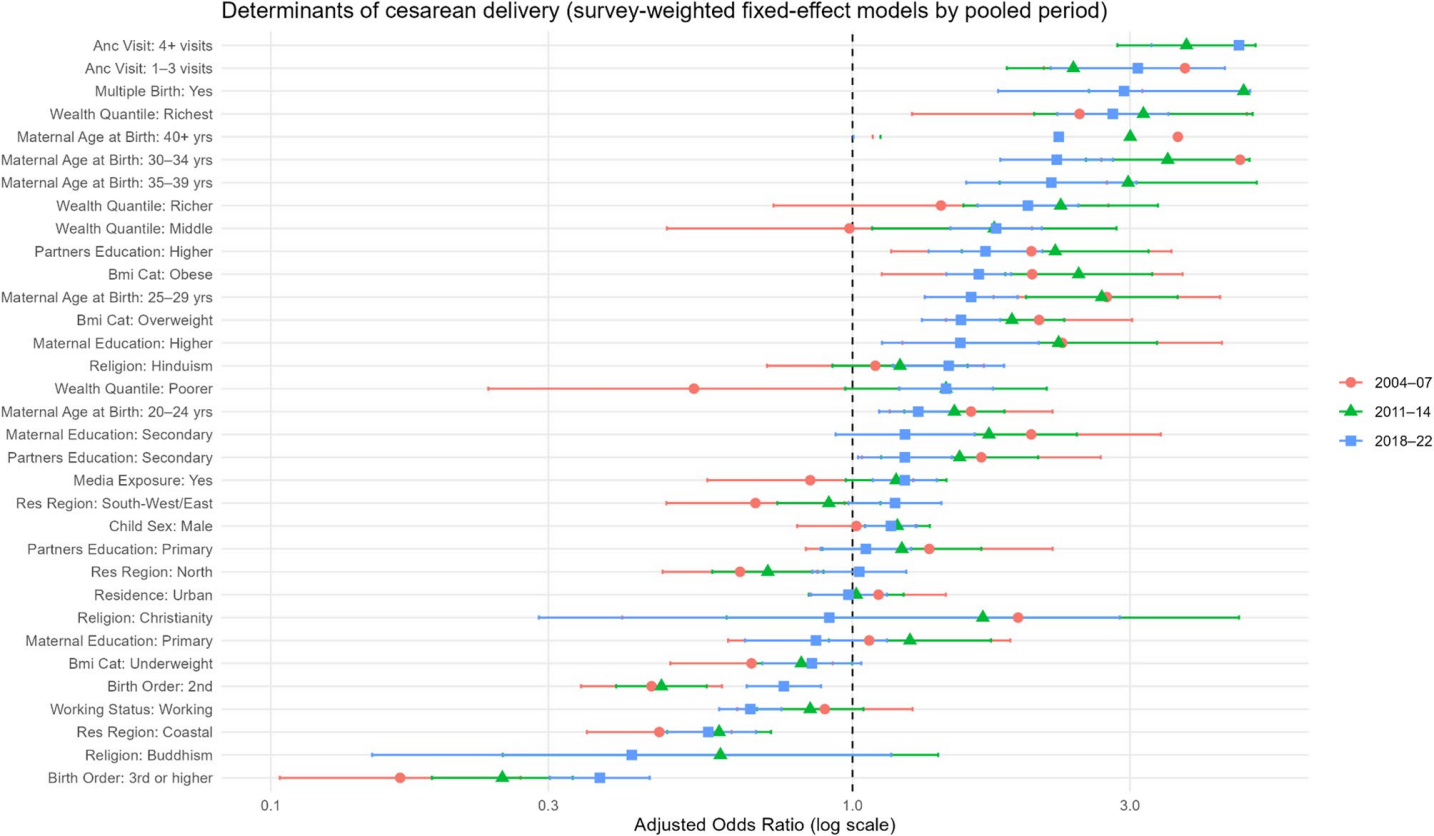
Determinants of caesarean delivery (survey-weighted fixed-effect models by pooled period), Bangladesh DHS 2004–2022.

The final analytic sample comprised a total of 26,845 ever-married women with a live birth in the three years preceding each survey. This included 7,278 births in the 2004–2007 period, 9,406 in 2011–2014, and 10,161 in 2018–2022. Table 1 presents the sociodemographic and healthcare characteristics of the study population across these three pooled periods, highlighting the shifting composition of mothers over time. To visualize the spatial and temporal evolution of caesarean section (CS) delivery, Fig. 1 maps the regional prevalence trends across the country. Furthermore, Fig. 2 illustrates the widening disparities by tracking prevalence trends across key sociodemographic subgroups (wealth, education, and antenatal care), while Fig. 3; Table 3 visualize and summarize the adjusted associations derived from the survey-weighted multivariable logistic regression models, identifying the independent factors of surgical delivery. The following subsections detail these patterns, examining the rapid rise in prevalence and the distinct factors fueling this increase.

**Table 1:** Distribution of characteristics by cesarean delivery status across pooled survey periods, Bangladesh DHS 2004–2022.

Characteristic	2004–07		2011–14		2018–22	
	No *N* = 6785	Yes *N* = 493	No *N* = 7470	Yes *N* = 1936	No *N* = 6201	Yes *N* = 3960
Delivery place, % (n)						
Home	100% (6,212)***	0% (0)***	100% (6,239)***	0% (0)***	100% (4,346)***	0% (0)***
Public sector	66% (357)***	34% (184)***	59% (689)***	41% (476)***	64% (1,021)***	36% (570)***
Private sector	34% (147)***	66% (280)***	21% (382)***	79% (1,400)***	16% (623)***	84% (3,279)***
NGO sector	63% (47)***	37% (28)***	71% (147)***	29% (59)***	61% (175)***	39% (112)***
Other	100% (20)***	0% (0)***	100% (14)***	0% (0)***	100% (36)***	0% (0)***
Multiple birth, % (n)						
No	93% (6,743)***	7% (481)***	80% (7,424)***	20% (1,910)***	61% (6,154)***	39% (3,906)***
Yes	78% (42)***	22% (12)***	64% (46)***	36% (26)***	46% (46)***	54% (54)***
Child sex, % (n)						
Female	93% (3,378)*	6.7% (241)*	80% (3,652)*	20% (888)*	62% (3,055)*	38% (1,846)*
Male	93% (3,406)*	7% (251)*	78% (3,818)*	22% (1,048)*	60% (3,146)*	40% (2,114)*
Birth order, % (n)						
1st	88% (2,032)***	12% (274)***	72% (2,566)***	28% (1,001)***	54% (2,083)***	46% (1,782)***
2nd	93% (1,748)***	7% (138)***	79% (2,201)***	21% (600)***	57% (1,944)***	43% (1,451)***
3rd or higher	97% (3,004)***	3% (81)***	89% (2,703)***	11% (335)***	75% (2,174)***	25% (727)***
ANC visit, % (n)						
No visit	99.3% (2,887)***	0.7% (19)***	95% (2,427)***	5% (121)***	92% (745)***	8% (66)***
1–3 visits	95% (2,828)***	5% (141)***	81% (3,398)***	19% (801)***	68% (3,363)***	32% (1,591)***
4 + visits	76% (1,068)***	24% (333)***	62% (1,644)***	38% (1,015)***	48% (2,093)***	52% (2,304)***
Residence, % (n)						
Rural	96% (5,517)***	4% (249)***	84% (5,967)***	16% (1,140)***	65% (4,824)***	35% (2,616)***
Urban	84% (1,267)***	16% (244)***	65% (1,503)***	35% (796)***	51% (1,377)***	49% (1,344)***
Maternal age at birth, % (n)						
< 20 years	95% (2,193)**	5% (126)**	81% (2,386)**	19% (550)**	63% (1,691)**	37% (985)**
20–24 years	92% (2,213)**	8% (182)**	80% (2,549)**	20% (639)**	59% (1,966)**	41% (1,352)**
25–29 years	92% (1,301)**	8% (106)**	76% (1,535)**	24% (485)**	61% (1,492)**	39% (951)**
30–34 years	93% (705)**	7% (55)**	77% (685)**	23% (207)**	59% (767)**	41% (523)**
35–39 years	93% (282)**	7% (21)**	84% (246)**	16% (48)**	65% (242)**	35% (131)**
40 + years	98% (90)**	2% (2)**	90% (69)**	10% (7)**	70% (42)**	30% (18)**
BMI, % (n)						
Normal	93% (3,846)***	7% (282)***	81% (4,499)***	19% (1,052)***	66% (3,029)***	34% (1,529)***
Underweight	97% (2,568)***	3% (74)***	88% (2,183)***	12% (285)***	74% (845)***	26% (297)***
Overweight	70% (262)***	30% (112)***	58% (612)***	42% (442)***	49% (715)***	51% (738)***
Obese	82% (108)***	18% (24)***	53% (175)***	47% (157)***	54% (1,611)***	46% (1,397)***
Maternal education, % (n)						
No education	99% (2,164)***	1% (27)***	94% (1,407)***	6% (90)***	80% (472)***	20% (121)***
Primary	98% (2,169)***	2% (52)***	89% (2,448)***	11% (288)***	77% (1,962)***	23% (590)***
Secondary	89% (2,160)***	11% (256)***	75% (3,262)***	25% (1,085)***	60% (3,131)***	40% (2,123)***
Higher	65% (291)***	35% (157)***	43% (352)***	57% (472)***	36% (635)***	64% (1,126)***
Partners education, % (n)						
No education	99% (2,601)***	1% (35)***	93% (2,251)***	7% (169)***	78% (1,123)***	22% (321)***
Primary	97% (1,930)***	3% (65)***	87% (2,442)***	13% (372)***	72% (2,294)***	28% (891)***
Secondary	90% (1,695)***	10% (183)***	74% (2,150)***	26% (745)***	58% (2,009)***	42% (1,478)***
Higher	73% (551)***	27% (208)***	49% (620)***	51% (649)***	36% (678)***	64% (1,226)***
Wealth index, % (n)						
Poorest	99% (1,659)***	1% (22)***	95% (1,990)***	4.8% (101)***	82% (1,712)***	18% (376)***
Poorer	99% (1,490)***	1% (18)***	90% (1,643)***	10% (183)***	70% (1,475)***	30% (630)***
Middle	97% (1,406)***	3% (44)***	83% (1,522)***	17% (307)***	61% (1,241)***	39% (781)***
Richer	93% (1,255)***	7.2% (97)***	73% (1,382)***	27% (503)***	54% (1,095)***	46% (935)***
Richest	76% (974)***	24% (312)***	53% (933)***	47% (841)***	35% (678)***	65% (1,238)***
Media exposure, % (n)						
No	98% (2,450)***	2% (52)***	91% (3,183)***	9% (306)***	74% (2,906)***	26% (1,044)***
Yes	91% (4,335)***	9% (441)***	72% (4,287)***	28% (1,630)***	53% (3,295)***	47% (2,916)***
Religion, % (n)						
Islam	93% (6,260)	7% (441)	80% (6,868)	20% (1,736)	62% (5,785)***	38% (3,583)***
Hinduism	90% (467)	10% (50)	74% (517)	26% (182)	50% (358)***	50% (359)***
Other	96% (57)	4% (2)	83% (85)	17% (17)	76% (58)***	24% (18)***
Working status, % (n)						
Not working	93% (5,421)***	7% (418)***	79% (6,261)***	21% (1,661)***	57% (4,101)***	43% (3,037)***
Working	95% (1,363)***	5% (74)***	81% (1,207)***	19% (275)***	69% (2,100)***	31% (924)***
Geographical region, % (n)						
Central	90% (2,057)***	10% (220)***	74% (2,296)***	26% (800)***	52% (1,321)***	48% (1,223)***
Coastal	95% (1,914)***	5% (98)***	84% (2,215)***	16% (435)***	71% (1,979)***	29% (821)***
North	95% (2,122)***	5% (113)***	83% (1,695)***	17% (357)***	60% (1,209)***	40% (792)***
South-West/East	92% (691)***	8% (62)***	79% (1,265)***	21% (344)***	55% (1,160)***	45% (943)***

***P-value<0.001, **P-value<0.01, *P-value<0.05; percentages are (approximately) rounded to the nearest integer.

### Facility distribution of surgical births

Between 2004 and 07 and 2018–22, the private sector became the primary driver of institutional deliveries and surgical births (Table 2). In the 2018–22 period, private facilities accounted for the vast majority of institutional deliveries and performed the highest proportion of all facility-based Caesarean sections, with an internal CS rate significantly exceeding that of public and NGO facilities.

**Table 2:** Share of institutional deliveries and CS by facility type.

Period	Facility type	% of inst. deliveries (*n*)	% of inst. CS (*n*)	CS rate % (95% CI)
2004-07	Public	50.7% (*n* = 541)	37.3% (*n* = 184)	34.0% (29.5–38.6)
	Private	40.0% (*n* = 427)	56.9% (*n* = 280)	65.7% (61.0-70.3)
	NGO	7.1% (*n* = 76)	5.8% (*n* = 28)	37.5% (26.1–48.9)
	Other	2.1% (*n* = 22)	0.0% (*n*= 0)	0.0% (0.0–0.0)
2011-14	Public	36.8% (*n* = 1,164)	24.6% (*n*= 476)	40.8% (37.3–44.4)
	Private	56.2% (*n* = 1,781)	72.3% (*n* = 1,400)	78.6% (76.4–80.8)
	NGO	6.5% (*n*= 206)	3.1% (*n*= 59)	28.7% (21.1–36.3)
	Other	0.5% (*n*= 16)	0.1% (*n* = 2)	9.7% (-4.1-23.5)
2018-22	Public	27.4% (*n* = 1,590)	14.4% (*n*= 570)	35.8% (33.0-38.6)
	Private	67.1% (*n* = 3,902)	82.8% (*n* = 3,279)	84.0% (82.5–85.6)
	NGO	4.9% (*n*= 287)	2.8% (*n*= 112)	38.9% (32.1–45.8)
	Other	0.6% (*n*= 36)	0.0% (*n* = 0)	0.0% (0.0–0.0)

### Trends in caesarean section prevalence and geographical variation

The prevalence of caesarean section (CS) delivery in Bangladesh exhibited a rapid and sustained escalation over the nearly two-decade study period. The overall weighted prevalence rose from 6.8% in the 2004–2007 pooled period to 20.6% in 2011–2014, reaching 39.0% in the most recent period of 2018–2022. This trajectory is further evidenced by annual estimates, which show rates climbing from approximately 5% in 2004 to nearly 45% by 2022 (Fig. 2A). This increasing trajectory was observed across all administrative areas, though marked geographical disparities persisted (Fig. 1). The Central region consistently reported the highest prevalence, rising from 9.7% in 2004–2007 to 48.1% in the 2018–2022 pooled period. In contrast, while the Coastal region also experienced a substantial relative increase, reaching 29.3% in the latest pooled estimates, it remained the region with the lowest prevalence throughout the study period.

Residential trends further highlight these disparities. As illustrated in Supplementary Figure S1, urban areas maintained consistently higher CS rates, starting at approximately 14% in 2004 and rising to over 50% by 2022. Aggregated over the survey waves, this resulted in a pooled prevalence of 49% for urban mothers in 2018–2022, compared to 35% for rural mothers (Table 1), confirming a widening yet persistent gap.

### Socioeconomic and health system inequalities

Disaggregated analysis revealed profound socioeconomic gradients in delivery modes, with inequalities widening significantly over time. Figure 2B demonstrates a clear divergence in CS prevalence by household wealth. In 2004, the prevalence among the richest quintile was approximately 18%, which surged to a pooled estimate of 65% in the 2018–2022 period (Table 1). In contrast, while the poorest quintile also saw an increase, their rates rose from near-zero levels (< 1%) in 2004 to just 18% in the most recent pooled period (Table 1), illustrating a massive expansion of the inequality gap. A similar pattern of divergence was observed for maternal education (Fig. 2C). Among women with higher education, CS rates climbed from approximately 30% in 2004 to a pooled prevalence of 64% in 2018–2022 (Table 1). Conversely, women with no formal education experienced a much slower rise, starting at roughly 2% in 2004 and reaching 20% in the final pooled period.

Facility type played a dominant structural role in these trends (Supplementary Figure S2). The private sector consistently exhibited the highest intensity of surgical delivery across all survey years. Starting at approximately 55% in 2004, the CS rate in private facilities followed a steep upward trajectory, reaching a pooled prevalence of 84% in 2018–2022 (Table 1). In comparison, public sector CS rates displayed a much flatter trend, starting at around 30% in 2004 and increasing only modestly to 36% in the most recent pooled period. Furthermore, antenatal care (ANC) utilization showed a strong dose-response relationship that persisted over time (Fig. 2D). The prevalence among women with 4 + ANC visits rose sharply from approximately 20% in 2004 to a pooled average of 52% in 2018–2022 (Table 1), whereas women with no ANC visits saw minimal change, remaining consistently below 10% throughout the study period.

**Table 3:** Survey-weighted adjusted odds ratios (AOR) with 95% confidence intervals and p-values for cesarean delivery, Bangladesh DHS 2004–2022.

Characteristic	2004–07			2011–14			2018–22		
	AOR	95% CI	*p*-value	AOR	95% CI	*p*-value	AOR	95% CI	*p*-value
Multiple birth									
No									
Yes	9.55	3.14–29.03	< 0.001	4.64	2.50–8.63	< 0.001	2.93	1.78–4.83	< 0.001
Child sex									
Female									
Male	1.02	0.80–1.28	0.894	1.19	1.05–1.36	0.007	1.16	1.05–1.29	0.003
Birth order									
1st									
2nd	0.45	0.34–0.60	< 0.001	0.47	0.39–0.56	< 0.001	0.76	0.66–0.88	< 0.001
3rd or higher	0.17	0.10–0.27	< 0.001	0.25	0.19–0.33	< 0.001	0.37	0.30–0.45	< 0.001
ANC visit									
No visit									
1–3 visits	3.73	2.13–6.54	< 0.001	2.40	1.85–3.13	< 0.001	3.10	2.20–4.38	< 0.001
4 + visits	9.62	5.26–17.62	< 0.001	3.76	2.86–4.94	< 0.001	4.62	3.27–6.54	< 0.001
Residence									
Rural									
Urban	1.11	0.85–1.45	0.448	1.02	0.84–1.23	0.842	0.99	0.85–1.15	0.858
Maternal age at birth									
< 20 years									
20–24 years	1.60	1.16–2.21	0.005	1.49	1.23–1.82	< 0.001	1.30	1.11–1.52	< 0.001
25–29 years	2.75	1.75–4.31	< 0.001	2.67	1.98–3.61	< 0.001	1.60	1.33–1.92	< 0.001
30–34 years	4.65	2.68–8.07	< 0.001	3.46	2.50–4.79	< 0.001	2.24	1.79–2.80	< 0.001
35–39 years	6.56	2.71–15.84	< 0.001	3.04	1.85–5.01	< 0.001	2.18	1.56–3.06	< 0.001
40 + years	3.64	1.09–12.21	0.036	2.99	1.11–8.06	0.030	2.27	1.01–5.10	0.046
BMI									
Normal									
Underweight	0.67	0.49–0.93	0.016	0.82	0.67–1.00	0.050	0.85	0.70–1.04	0.107
Overweight	2.09	1.45–3.03	< 0.001	1.88	1.52–2.31	< 0.001	1.54	1.32–1.80	< 0.001
Obese	2.03	1.12–3.70	0.020	2.44	1.83–3.26	< 0.001	1.65	1.45–1.87	< 0.001
Maternal education									
No education									
Primary	1.08	0.62–1.89	0.789	1.26	0.91–1.73	0.166	0.87	0.65–1.15	0.315
Secondary	2.05	1.23–3.43	0.006	1.72	1.21–2.43	0.002	1.23	0.94–1.62	0.136
Higher	2.31	1.23–4.36	0.010	2.27	1.54–3.35	< 0.001	1.53	1.12–2.09	0.007
Partners education									
No education									
Primary	1.35	0.83–2.21	0.226	1.22	0.89–1.67	0.223	1.05	0.88–1.26	0.566
Secondary	1.67	1.04–2.68	0.035	1.53	1.12–2.09	0.008	1.23	1.02–1.48	0.028
Higher	2.03	1.16–3.54	0.013	2.23	1.54–3.23	< 0.001	1.69	1.35–2.12	< 0.001
Wealth index									
Poorest									
Poorer	0.53	0.23–1.20	0.127	1.45	0.97–2.16	0.070	1.45	1.20–1.74	< 0.001
Middle	0.98	0.48–2.03	0.963	1.75	1.08–2.84	0.023	1.76	1.47–2.11	< 0.001
Richer	1.41	0.73–2.73	0.311	2.28	1.55–3.34	< 0.001	2.00	1.64–2.44	< 0.001
Richest	2.44	1.26–4.74	0.009	3.16	2.05–4.88	< 0.001	2.79	2.24–3.48	< 0.001
Media exposure									
No									
Yes	0.85	0.56–1.28	0.431	1.19	0.97–1.45	0.089	1.23	1.08–1.40	0.001
Religion									
Islam									
Hinduism	1.09	0.71–1.68	0.680	1.21	0.92–1.58	0.168	1.46	1.17–1.82	< 0.001
Other	1.48	0.36–6.05	0.582	0.78	0.38–1.59	0.488	0.55	0.26–1.14	0.107
Working status									
Not working									
Working	0.89	0.63–1.27	0.525	0.85	0.69–1.04	0.120	0.67	0.59–0.75	< 0.001
Geographic region									
Central									
Coastal	0.46	0.35–0.61	< 0.001	0.59	0.48–0.72	< 0.001	0.56	0.47–0.68	< 0.001
North	0.64	0.47–0.87	0.004	0.71	0.57–0.89	0.003	1.03	0.85–1.24	0.774
South-West/East	0.68	0.48–0.97	0.033	0.91	0.74–1.12	0.370	1.19	0.99–1.43	0.068

### Determinants of caesarean delivery

After adjusting for potential confounders in survey-weighted multivariable logistic regression models, distinct shifts in the socioeconomic and clinical drivers of caesarean section (CS) emerged across the three study periods (Table 3). A visual inspection of the forest plot (Fig. 3) reveals how the strength and direction of these associations have evolved, highlighting a transition where surgical delivery has permeated from the most affluent to the broader population.

### Socioeconomic factors

Household wealth status remained a potent driver throughout the two decades, but the dynamic between wealth groups shifted notably. As evident in Fig. 3, the gap has widened to include the lower-middle class. In the 2004–2007 period, women in the poorer wealth quintile were actually protective against CS, being 47% less likely to undergo the procedure compared to the poorest women (AOR 0.53; 95% CI 0.23–1.20). However, by 2018–2022, this relationship reversed entirely; women in the poorer quintile were now 45% more likely to deliver via CS than the poorest group (AOR 1.45; 95% CI 1.20–1.74). This graduation of risk suggests that financial barriers to accessing CS are lowering even for households with modest incomes. Meanwhile, the richest quintile consistently maintained the highest odds (AOR 2.80 in 2018–2022), though the exclusivity of CS to the socio-cultural elite appears to be diminishing.

This widespread saturation effect is particularly evident in parental education. While higher education for both mothers and partners remained a statistically significant predictor across all waves (*p* < 0.01), the magnitude of its influence has faded markedly over time. The adjusted odds for women with higher education dropped from 2.31 (95% CI 1.23–4.36) in 2004–2007 to 1.53 (95% CI 1.12–2.09) in 2018–2022. A similar tendency was observed for partners with higher education, where the odds ratio decreased from 2.03 in the earliest period to 1.69 in recent years. This downward trend suggests that surgical delivery is increasingly overcoming educational boundaries, becoming a generalized practice rather than a privilege of the highly educated.

### Healthcare and clinical factors

The forest plot shows notable clinical risk saturation; ANC visits remain the top non-biological predictor, though their impact has decreased considerably over time. In 2004–2007, women with four or more ANC visits faced a staggering nearly ten-fold increase in odds (AOR 9.62; 95% CI 5.26–17.62). By 2018–2022, while still the dominant predictor, the odds ratio had more than halved to 4.62 (95% CI 3.27–6.53). A similar downward trend is visible for multiple births, a distinct medical indication. The odds of CS for multiple pregnancies dropped from 9.55 in the earliest period to 2.93 in recent years. This reduction across clinical factors suggests that as CS rates rise globally, the procedure is increasingly being driven by non-medical factors rather than strictly by obstetric emergencies or high-intensity care demands.

### Biomedical and geographical risk factors

Dietary habits exhibited a comparable pattern of attenuation. While high BMI consistently elevated the risk of CS, the strength of this association has weakened over the study period. The odds of CS for overweight women decreased from 2.09 (95% CI 1.45–3.03) in the earliest period to 1.54 (95% CI 1.32–1.80) in recent years. Likewise, the elevated risk associated with obesity, which peaked at 2.44 in 2011–2014, declined to 1.65 (95% CI 1.45–1.87) in 2018–2022. Despite this reduction in odds, advanced maternal age remained a steadfast predictor; women aged 30–34 continued to have more than double the likelihood of CS in 2018–2022 (AOR 2.24). Geographically, the protective effect of living outside the central hub remained significant. Residents of the Coastal region consistently exhibited roughly half the odds of CS compared to those in the Central region across all three periods (AOR 0.57 in 2018–2022). Notably, once these socioeconomic and regional factors were accounted for, the urban advantage disappeared in the most recent models (AOR 0.99; *p* = 0.858), confirming that the higher rates in cities are driven by the concentration of wealth and private facilities rather than urban residence itself.

### Sensitivity analysis

A woman’s likelihood of undergoing a CS is strongly determined by her history of prior cesarean deliveries. Therefore, the results presented in the main analysis may be biased by the inclusion of multiparous women. To remove this potential clinical confounding, we conducted a sensitivity analysis using a restricted sample of first-order births only. Overall, similar patterns were observed; wealth quantile and ANC visits remained strongly associated with cesarean delivery in the restricted sample. For instance, in the 2018–22 period, women in the richest wealth quantile (aOR 2.24) and those with 4 + ANC visits (aOR 3.91) continued to show significantly higher odds of CS compared to their reference groups. However, we observed unstable estimates with extremely wide confidence intervals for the maternal age 40 + category in this model; this was due to the very small number of primiparous women in this age group across the survey periods.

The main analysis included all deliveries regardless of place of birth, which may introduce bias because home births in Bangladesh are predominantly vaginal, potentially combining the lack of access to facility care with the effect of lower socioeconomic status. To address this issue and isolate predictors of CS, we estimated a second model restricted to facility births only. The results using this subsample were largely consistent with the main findings; the richest wealth quantile remained a significant predictor (aOR 1.95 in 2018–22), and higher birth order was significantly associated with lower odds of cesarean delivery compared to first births. These additional analyses confirm that the associations between socioeconomic factors and cesarean delivery reported in this study are robust to sample specification and not solely driven by repeat CS or lack of facility access [Supplementary Material].

### Sensitivity analysis: institutional deliveries only

**Fig. 4:**
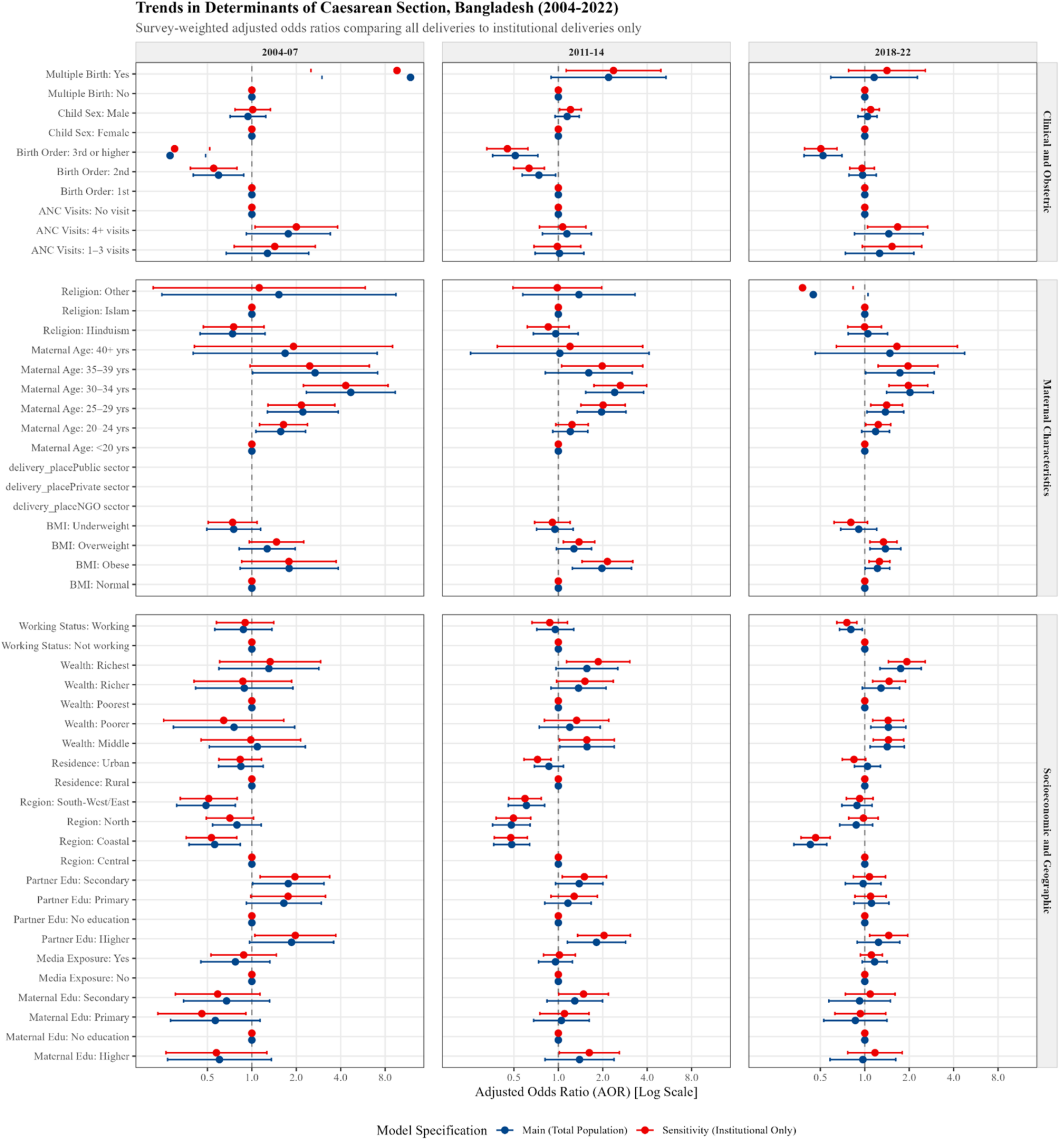
Trends in determinants of Caesarean section in Bangladesh (2004–2022): A comparison of total population and institutional-only deliveries. Forest plot displaying survey-weighted adjusted odds ratios (AOR) and 95% confidence intervals (CI) across three pooled periods. Blue markers represent the main multivariable logistic regression model encompassing all recent births. Red markers represent the sensitivity analysis restricted exclusively to women who delivered in a health facility (excluding home births). Both models adjust for the displayed socioeconomic, maternal, and clinical covariates; the sensitivity model intentionally omits ‘place of delivery’ to avoid circularity. Points situated exactly at the dashed line (AOR = 1.0) denote the reference category for each respective variable.

We limited our models to institutional deliveries to make sure that relations observed, especially those related to wealth and education, were not simply due to differences in access to health facilities (see Table 4; Fig. 4). The sensitivity analysis confirmed that socioeconomic gaps were still present among women who made it to a health facility. Overall, the direction of associations remained consistent with the main models. Household wealth continued to be positively associated with CS delivery within facilities, with women in the richest quintile exhibiting higher odds compared to the poorest group in 2018–2022 (OR 1.95; 95% CI 1.46–2.60). Also, higher maternal and partner education were both positively associated with CS delivery within institutional settings across pooled periods. Consistent with the primary analysis, higher birth order was associated with lower odds of CS across all periods, while maternal age and overweight status retained positive associations with surgical delivery in this subsample.

**Table 4:** Sensitivity analysis restricted to institutional deliveries.

Characteristic	2004-07		2011-14		2018-22	
	OR (95% CI)	*p*-value	OR (95% CI)	*p*-value	OR (95% CI)	*p*-value
Wealth index						
Poorest	–		–		–	
Poorer	0.64 (0.25–1.64)	0.36	1.33 (0.81–2.20)	0.27	1.44 (1.13–1.83)	0.003
Middle	0.98 (0.45–2.14)	0.97	1.56 (1.02–2.39)	0.042	1.45 (1.14–1.83)	0.002
Richer	0.87 (0.41–1.86)	0.72	1.51 (0.97–2.36)	0.066	1.46 (1.13–1.89)	0.004
Richest	1.33 (0.61–2.93)	0.48	1.86 (1.14–3.06)	0.014	1.93 (1.45–2.57)	< 0.001
Maternal education						
No education	–		–		–	
Primary	0.46 (0.23–0.91)	0.026	1.10 (0.75–1.61)	0.62	0.93 (0.63–1.39)	0.73
Secondary	0.59 (0.30–1.14)	0.11	1.48 (1.01–2.19)	0.047	1.09 (0.74–1.60)	0.67
Higher	0.58 (0.26–1.26)	0.17	1.62 (1.01–2.59)	0.043	1.17 (0.77–1.79)	0.46
Partners education						
No education	–		–		–	
Primary	1.76 (0.98–3.15)	0.057	1.28 (0.89–1.84)	0.18	1.09 (0.86–1.40)	0.47
Secondary	1.96 (1.14–3.37)	0.015	1.50 (1.06–2.12)	0.021	1.08 (0.84–1.38)	0.57
Higher	1.97 (1.05–3.70)	0.036	2.04 (1.35–3.08)	< 0.001	1.45 (1.08–1.96)	0.014
Residence						
Rural	–		–		–	
Urban	0.83 (0.60–1.16)	0.28	0.72 (0.59–0.89)	0.002	0.84 (0.70–1.01)	0.070
ANC visit						
No visit	–		–		–	
1–3 visits	1.43 (0.76–2.69)	0.27	0.98 (0.68–1.42)	0.94	1.53 (0.96–2.43)	0.072
4 + visits	2.00 (1.05–3.81)	0.035	1.07 (0.74–1.54)	0.71	1.67 (1.04–2.67)	0.032
Multiple birth						
No	–		–		–	
Yes	9.63 (2.51–36.9)	0.001	2.37 (1.13–4.96)	0.023	1.41 (0.78–2.57)	0.26
Child Sex						
Female	–		–		–	
Male	1.01 (0.77–1.34)	0.92	1.21 (1.02–1.43)	0.027	1.10 (0.96–1.25)	0.18
BMI						
Normal	–		–		–	
Underweight	0.74 (0.51–1.08)	0.12	0.91 (0.69–1.20)	0.51	0.80 (0.62–1.04)	0.10
Overweight	1.47 (0.96–2.25)	0.076	1.38 (1.08–1.77)	0.010	1.34 (1.08–1.65)	0.007
Obese	1.78 (0.85–3.73)	0.12	2.15 (1.45–3.20)	< 0.001	1.26 (1.07–1.48)	0.006
Geographic region						
Central	–		–		–	
Coastal	0.53 (0.36–0.79)	0.002	0.48 (0.37–0.62)	< 0.001	0.46 (0.37–0.58)	< 0.001
North	0.71 (0.49–1.03)	0.069	0.50 (0.38–0.65)	< 0.001	0.98 (0.78–1.23)	0.85
South-West/East	0.51 (0.33–0.80)	0.003	0.59 (0.46–0.77)	< 0.001	0.92 (0.75–1.14)	0.45
Maternal age at birth						
< 20 years	–		–		–	
20–24 years	1.64 (1.13–2.38)	0.010	1.24 (0.96–1.59)	0.10	1.23 (1.01–1.50)	0.035
25–29 years	2.17 (1.29–3.65)	0.004	2.01 (1.42–2.84)	< 0.001	1.41 (1.10–1.80)	0.007
30–34 years	4.33 (2.24–8.38)	< 0.001	2.63 (1.74–3.97)	< 0.001	1.97 (1.46–2.67)	< 0.001
35–39 years	2.47 (0.97–6.26)	0.057	1.98 (1.05–3.74)	0.034	1.96 (1.23–3.13)	0.005
40 + years	1.92 (0.41–8.99)	0.41	1.20 (0.39–3.73)	0.75	1.65 (0.64–4.24)	0.30
Media exposure						
No	–		–		–	
Yes	0.88 (0.53–1.46)	0.62	1.02 (0.79–1.30)	0.89	1.11 (0.93–1.32)	0.24
Religion						
Islam	–		–		–	
Hinduism	0.75 (0.47–1.21)	0.24	0.85 (0.62–1.18)	0.35	1.00 (0.77–1.30)	0.98
Other	1.12 (0.21–5.86)	0.89	0.98 (0.49–1.97)	0.96	0.38 (0.17–0.83)	0.016
Working status						
Not working	–		–		–	
Working	0.90 (0.58–1.41)	0.64	0.87 (0.66–1.15)	0.34	0.76 (0.65–0.88)	< 0.001
Birth order						
1st	–		–		–	
2nd	0.55 (0.38–0.79)	0.001	0.63 (0.50–0.80)	< 0.001	0.96 (0.79–1.16)	0.66
3rd or higher	0.30 (0.17–0.52)	< 0.001	0.45 (0.33–0.62)	< 0.001	0.50 (0.39–0.65)	< 0.001

Abbreviations: CI = confidence interval, OR = odds ratio.

## Discussion

This study provides a comprehensive, twenty-year assessment of caesarean section (CS) trends in Bangladesh, revealing a landscape where surgical delivery has evolved from a specialized medical intervention into a dominant, private-sector-driven mode of childbirth. Our analysis illuminates a dramatic escalation in CS prevalence—rising from roughly 5% in 2004 to 45% in 2022. This upward trajectory is consistent with the findings of recent BDHS-based studies, which documented average annual increases of 13–19% in earlier periods, confirming that the surge has continued relentlessly^19,29^. While our BDHS-based pooled estimate of 39% for the most recent period is lower than the 67.4% prevalence reported using MICS 2019 data by other national surveys, both datasets confirm that Bangladesh has far exceeded the World Health Organization’s recommended threshold of 10–15%, positioning the country within the critical South Asian anomaly of rising surgical births alongside neighbors like Nepal and Pakistan^30^.

One of the primary objectives of this research was to determine the role of healthcare systems in driving this increase. Our results identify the private sector as the engine to this growth, with CS rates in private facilities reaching a staggering 84% in the most recent period. This finding upholds a wealth of existing literature; recent research reported that private hospital delivery increased CS odds by nearly 39 times, while earlier global health analyses observed similar private-sector dominance (73%) nearly a decade ago^31,32^. The persistence of these high rates supports the provider-induced demand hypothesis suggested by previous literature, noting that external agents and private providers often receive financial rewards for surgical deliveries, creating a conflict of interest that overrides evidence-based protocols^33^. Unlike the public sector, where rates have remained closer to medically justifiable levels, the private sector appears to operate under a different logic, where surgery is marketed as a modern and convenient service, effectively normalizing the procedure among urban and affluent populations^34^. The overwhelming shift toward private facilities, despite the availability of free or heavily subsidized public maternity services, suggests a complex demand-side dynamic. This trend is driven by several reasons: people believe private facilities provide better quality care, friendlier communication, and more flexible scheduling. Additionally, fears about labor pain and social beliefs that a surgical birth is safer or more prestigious encourage women to opt for private clinics, where provider-initiated CS is more readily accommodated^35^.

The institutionalization of this practice is further evidenced by the significant socioeconomic and geographic disparities identified throughout our research. We found that women in the richest wealth quintile were nearly three times as likely to undergo CS as those in the poorest quintile. This aligns with the inverse equity hypothesis described in recent inequality analyses, which highlighted a double burden: overuse among the affluent and underuse among the disadvantaged^29^. However, this inequality has a distinct spatial dimension. The concentration of surgical deliveries in the Central region and urban hubs, where the urban advantage dissipated after controlling for wealth, suggests that geography acts as a proxy for access to the unregulated private market.

Our forest plot analysis adds a subtle layer to this narrative: the “risk gap” is narrowing for the middle class, suggesting that as economic stability improves, the burden of overuse is streaming down from the elite to the broader population. This democratization of surgical birth is likely driven by the aspirations of the emerging middle class to access what is perceived as superior care, a trend previously identified in resource-poor settings^36^.

A particularly notable finding regarding the shift toward private-sector dominance is the strong and persistent association between antenatal care (ANC) utilization and caesarean delivery. While ANC is designed to safeguard maternal health, our data reveals a dose-response relationship where women with four or more visits are over four times more likely to deliver via CS. This confirms the ANC Paradox observed in recent empirical evidence, which found that women with ANC access had over four times higher odds of elective CS^37^. M. Pulok et al. (2016) and Jahar Bhowmik et al. (2019) have similarly linked frequent ANC visits to higher intervention rates^38,39^. This suggests that in the current Bangladeshi context, increased contact with the medical system often serves as a “recruitment pathway” for surgical delivery rather than a mechanism for promoting physiological birth. This may be due to risk labeling during routine check-ups or the influence of providers who favor scheduled deliveries^40^.

Finally, the question of whether CS remains a life-saving intervention is challenged by the shifting risk profile. Our finding that advanced maternal age and obesity are independent predictors aligns with prior findings, which attributed a significant portion of the CS burden to rising rates of maternal overweight/obesity^41^. However, the attenuation of odds for traditional clinical indicators (like multiple births) in our study suggests that medical necessity is increasingly being overshadowed by non-clinical drivers. As noted in the literature, when providers ignore evidence-based guidelines, the decision for surgery becomes subjective, driven by fear of labor pain or institutional financial incentives rather than obstetric emergency^33,40^.

In summary, the rapid rise of CS in Bangladesh is a multi-factorial phenomenon driven by unregulated private sector expansion, socioeconomic transitions, and a medical culture that incentivizes intervention. The alignment of our findings with established health systems research underscores that without systemic reform, this trajectory threatens to divert critical resources away from essential maternal care, hindering the nation’s progress toward equitable health outcomes^5,42^.

## Strengths and limitations

To our knowledge, this is one of the most comprehensive studies utilizing pooled data from all the relevant BDHS survey years to examine the long-term evolution of caesarean section determinants in Bangladesh. The use of survey-weighted models and sensitivity analyses strengthens the validity of our findings regarding the socioeconomic and facility-level drivers of surgical birth. However, several limitations should be considered when interpreting these results.

First, the cross-sectional nature of the BDHS design precludes the establishment of causal inferences between sociodemographic factors and the decision to undergo CS. While we observed strong associations, such as the link between antenatal care frequency and surgical delivery, we cannot definitively determine the direction of causality or rule out unmeasured confounders, such as the specific content of counseling received during ANC visits.

Second, the dataset lacks detailed clinical information necessary to definitively distinguish between medically indicated (emergency) and non-indicated (elective) caesarean sections. Without access to medical charts or variables required for the Robson Ten Group Classification System (such as detailed labor induction history or fetal heart rate patterns), we relied on sociodemographic proxies to infer the drivers of the rising CS rate^43^. Consequently, while the high prevalence among low-risk groups suggests a rise in non-medically indicated procedures, we could not quantify the exact proportion of maternal requests versus provider-initiated surgeries.

Third, our categorization of delivery facilities was limited to a binary public vs. private distinction due to the structure of the survey data. This broad categorization obscures the significant heterogeneity within Bangladesh’s private sector, which ranges from high-quality tertiary care hospitals to smaller, unregulated clinics. Identifying which specific tier of private facilities is driving the surge in CS rates was beyond the scope of this analysis but remains a critical area for future health systems research.

Fourth, as with all DHS-based studies, the data are subject to recall bias, as information regarding delivery complications and decision-making was self-reported by mothers up to three years after the birth. Furthermore, as highlighted in our sensitivity analysis, sample size constraints resulted in unstable estimates for specific subgroups, particularly primiparous women of late maternal age (40 + years). While this did not affect the overall trends, it limited our ability to draw precise conclusions for this specific demographic.

Finally, while we adjusted for a wide range of covariates, the private sector effect observed in our models may still be partially confounded by unmeasured factors, such as the varying availability of anesthesiology services or the specific financial incentives of individual providers, which are not captured in household surveys. Despite these limitations, the consistency of our findings across sensitivity analyses, including models restricted to facility-based births and first-order births, suggests that the identified trends are robust and reflect a genuine systemic shift in Bangladesh’s obstetric culture.

## Policy recommendations

The rapid rise of caesarean delivery in Bangladesh, largely driven by the private sector, necessitates urgent policy reform focused on quality assurance and accountability. We recommend implementing mandatory clinical audits and transparent reporting across all facilities to distinguish medically indicated procedures from avoidable ones. Concurrently, health systems must decouple financial incentives from surgical decision-making to address the dual burden of supply-induced overuse and inequitable underuse. To reverse this trend, systemic efforts must focus on improving the attractiveness and utilization of public sector maternity services. This includes ensuring 24/7 availability of emergency obstetric care, enhancing the physical infrastructure of public labor wards to ensure privacy, and deploying midwifery-led continuity of care models that provide respectful, patient-centered support to reduce fear of vaginal birth. Finally, strengthening respectful maternity care and unbiased counseling during antenatal visits is essential to empower women, ensuring that surgical delivery remains a life-saving intervention rather than a routine practice.

## Conclusion

Caesarean section (CS) delivery in Bangladesh has risen rapidly over the past two decades, reaching approximately 39% in the most recent pooled period. Delivery in a private facility, higher household wealth, advanced maternal age, and increased number of antenatal visits were significant predictors of CS. There were marked disparities in CS use across geographical locations and socioeconomic groups, with the Central region and urban areas consistently reporting higher prevalence than the Coastal and rural regions. The private sector emerged as the primary driver of this increase, with surgical rates exceeding 80%, suggesting that the procedure is increasingly being driven by non-medical factors rather than obstetric emergencies. The strong association between frequent antenatal care and CS indicates that contact with the healthcare system, particularly in unregulated settings, often predisposes women to surgical delivery.

These findings warrant further policy intervention to examine the provider-side incentives driving excessive use in the private sector and to assess whether the rapid rise among the middle class reflects genuine need or provider-induced demand. Rationalizing CS use is a complex task which requires strict regulatory oversight and comprehensive health systems reform. Service providers need to be better regulated through mandatory clinical audits to ensure that CS is only carried out when medically necessary and not for financial gains. At the same time, ensuring equitable access for the disadvantaged remains critical to prevent a double burden of overuse among the affluent and underuse among the poor.

## Supplementary Information

Below is the link to the electronic supplementary material.

Supplementary Material 1

## Data Availability

The data used in this study are publicly available from the Demographic and Health Surveys (DHS) Program upon reasonable request and approval. Access to the data can be obtained from https:/dhsprogram.com.

## Declarations

### Patient and public involvement

Patients and the public were not involved in the design, conduct, reporting, or dissemination plans of this research.

## References

[1] Betran, A. P. et al. What is the optimal rate of caesarean section at population level? A systematic review of ecologic studies. *Reprod. Health*. **12** (1), 57. 10.1186/s12978-015-0043-6 (2015). 26093498 10.1186/s12978-015-0043-6 PMC4496821

[2] Souza, J. et al. Caesarean section without medical indications is associated with an increased risk of adverse short-term maternal outcomes: the 2004–2008 WHO Global Survey on Maternal and Perinatal Health. *BMC Med.* **8** (1), 71. 10.1186/1741-7015-8-71 (2010). 21067593 10.1186/1741-7015-8-71 PMC2993644

[3] WHO statement on caesarean section rates [Internet]. [cited 2026 Jan 8]. Available from: https://www.who.int/publications/i/item/WHO-RHR-15.02.

[4] Cleveland Clinic [Internet]. [cited 2026 Jan 16]. C-Section (Cesarean Section): Procedure, Risks & Recovery. Available from: https://my.clevelandclinic.org/health/treatments/7246-cesarean-birth-c-section.

[5] Haider, M. R. et al. Ever-increasing Caesarean section and its economic burden in Bangladesh. *PLOS ONE*. **13** (12), e0208623. 10.1371/journal.pone.0208623 (2018). 30532194 10.1371/journal.pone.0208623 PMC6287834

[6] Hu, Y., Tao, H. & Cheng, Z. Caesarean Sections in Beijing, China - Results from a Descriptive Study. *Gesundheitswesen* **78** (1), e1–5. 10.1055/s-0035-1549937 (2016). PubMed PMID: 26140579. 26140579 10.1055/s-0035-1549937

[7] Caesarean section rates. continue to rise, amid growing inequalities in access [Internet]. [cited 2026 Jan 11]. Available from: https://www.who.int/news/item/16-06-2021-caesarean-section-rates-continue-to-rise-amid-growing-inequalities-in-access.

[8] Neuman, M. et al. Prevalence and determinants of caesarean section in private and public health facilities in underserved South Asian communities: cross-sectional analysis of data from Bangladesh, India and Nepal. *BMJ Open.* **4** (12), e005982. 10.1136/bmjopen-2014-005982 (2014). PubMed PMID: 25550293. 25550293 10.1136/bmjopen-2014-005982 PMC4283435

[9] Rana, M. S., Mazumder, S., Khan, M. T. F., Khan, M. M. H. & Rahman, M. M. Trends and determinants of caesarean section in South Asian countries: Bangladesh, Nepal, and Pakistan. *PLOS ONE*. **19** (12), e0311082. 10.1371/journal.pone.0311082 (2024). 39637169 10.1371/journal.pone.0311082 PMC11620693

[10] Kundu, S. et al. Socioeconomic and geographical inequalities in delivery by cesarean section among women in Bangladesh, 2004–2017. *BMC Pregnancy Childbirth*. **24**, 131. 10.1186/s12884-024-06327-z (2024). PubMed PMID: 38350916; PubMed Central PMCID: PMC10863140. 38350916 10.1186/s12884-024-06327-z PMC10863140

[11] Haque, M. R., Rabbi, A. M. F., Hasan, M. S., Araf, F. & Islam, M. S. Does community pressure matter in cesarean deliveries in Bangladesh? An analysis of nationally representative surveys. *PLOS ONE*. **20** (8), e0328162. 10.1371/journal.pone.0328162 (2025). 40828803 10.1371/journal.pone.0328162 PMC12364313

[12] Khan, M. N., Kabir, M. A., Shariff, A. A. & Rahman, M. M. Too many yet too few caesarean section deliveries in Bangladesh: Evidence from Bangladesh Demographic and Health Surveys data. *PLOS Global Public. Health*. **2** (2), e0000091. 10.1371/journal.pgph.0000091 (2022). 36962249 10.1371/journal.pgph.0000091 PMC10022004

[13] Angolile, C. M., Max, B. L., Mushemba, J. & Mashauri, H. L. Global increased cesarean section rates and public health implications: A call to action. *Health Sci. Rep.* **6** (5), e1274. 10.1002/hsr2.1274 (2023). 37216058 10.1002/hsr2.1274 PMC10196217

[14] Karim, F. S., Maharana, S., Akhter, S. & Chowdhury, S. R. Knowledge, attitude, practice, and perceived barriers to antenatal yoga among obstetricians and gynecologists in Bangladesh: A cross-sectional survey. *Complement. Ther. Clin. Pract.* **59**, 101981. 10.1016/j.ctcp.2025.101981 (2025). 40194465 10.1016/j.ctcp.2025.101981

[15] Zoha, S., Alam, S., Sifat, I. K., Sultana, N. & Kibria, M. K. Identifying determinants and predicting cesarean section delivery among Bangladeshi women using machine learning: Insight from BDHS 2022 Data. *PLOS Global Public. Health*. **5** (11), e0005494. 10.1371/journal.pgph.0005494 (2025). 41259389 10.1371/journal.pgph.0005494 PMC12629447

[16] Chowdhury, M. A. B., Adnan, M. M. & Hassan, M. Z. Trends, prevalence and risk factors of overweight and obesity among women of reproductive age in Bangladesh: A pooled analysis of five national cross-sectional surveys [Internet]. Jul **1**. 10.1136/bmjopen-2017-018468 (2018). 10.1136/bmjopen-2017-018468 PMC6059314 30030307

[17] Alem, A. Z. et al. Double burden of malnutrition and its associated factors among women in low and middle income countries: findings from 52 nationally representative data. *BMC Public. Health*. **23** (1), 1479. 10.1186/s12889-023-16045-4 (2023). 37537530 10.1186/s12889-023-16045-4 PMC10398981

[18] Sujon, M. S. H., Sumon, I. H., Ahmmad, J., Asif, M. S. & Hossain, M. M. Determinants of cesarean section in urban areas of Bangladesh: Insights from the Bangladesh Demographic and Health Survey-2022. *Womens Health (Lond)*. **21**, 17455057251356806 (2025). doi:10.1177/17455057251356806 PubMed PMID: 40785446; PubMed Central PMCID: PMC12340205. 40785446 10.1177/17455057251356806 PMC12340205

[19] Kjerulff, K. H., Zhu, J., Weisman, C. S. & Ananth, C. V. First birth Caesarean section and subsequent fertility: a population-based study in the USA, 2000–2008. *Hum. Reprod.* **28** (12), 3349–3357. 10.1093/humrep/det343 (2013). 24021550 10.1093/humrep/det343 PMC3829579

[20] Karmakar, G. et al. Region-specific variation and determinants of caesarean delivery among ever-married women in Bangladesh. *PLOS ONE*. **20** (9), e0328830. 10.1371/journal.pone.0328830 (2025). 40938870 10.1371/journal.pone.0328830 PMC12431222

[21] Geographical Disparities and Contributing Factors. Linked to Cesarean Section Deliveries in Bangladesh: Bangladesh Demographic and Health Survey 2017–2018 analysis.

[22] Khan, M. N., Alam, M. B., Khanam, S. J., Islam, M. M. & Billah, M. A. Trends, district-level variations, and socioeconomic disparities in cesarean section delivery in Bangladesh. *PLOS ONE*. **20** (10), e0334931. 10.1371/journal.pone.0334931 (2025). 41171712 10.1371/journal.pone.0334931 PMC12578250

[23] Kumar, P. & Sharma, H. Prevalence and determinants of socioeconomic inequality in caesarean section deliveries in Bangladesh: an. *BMC Pregnancy Childbirth*. **23**, 492. 10.1186/s12884-023-05782-4 (2023). PubMed PMID: 37403091; PubMed Central PMCID: PMC10320922analysis of cross-sectional data from Bangladesh Demographic Health Survey, 2017-18. 10.1186/s12884-023-05782-4 PMC10320922 37403091

[24] Turjo, E. A., Rahman, A., Noor, M. T. B. & Shakib, A. H. Factors influencing cesarean delivery in Bangladesh. *Discover Public. Health*. **22** (1), 1–15. 10.1186/s12982-025-00850-w (2025).

[25] Sarker, A., Chowdhury, S., Begum, M., Bhowmik, J. & Islam, M. Women’s fear of normal delivery and their decision on the mode of delivery: a cross-sectional study. *Int. J. Reprod. Contracept. Obstet. Gynecol.* **11**, 2426. 10.18203/2320-1770.ijrcog20222305 (2022).

[26] Causes and Complications of Cesarean Section. Delivery among Women in Cox’s Bazaar. *Bangladesh EJMHS*. 1–11. 10.34104/ejmhs.021.01011 (2021).

[27] Begum, T. et al. Indications and determinants of caesarean section delivery: Evidence from a population-based study in Matlab, Bangladesh. *PLOS ONE*. **12** (11), e0188074. 10.1371/journal.pone.0188074 (2017). 29155840 10.1371/journal.pone.0188074 PMC5695799

[28] Training, N. I., PR, Welfare, F. W., of H, M. & F, I. C. F. of and, Division ME and and Bangladesh Demographic and Health Survey 2022: Final Report [Internet]. 2024 Jan 15 [cited 2026 Feb 17]. Available from: https://dhsprogram.com/publications/publication-fr386-dhs-final-reports.cfm.

[29] The, D. H. S. Program - Demographic and Health Survey (DHS) [Internet]. [cited 2026 Feb 8]. Available from: https://dhsprogram.com/methodology/survey-Types/dHs.cfm.

[30] Khan, M. N., Islam, M. M., Shariff, A. A., Alam, M. M. & Rahman, M. M. Socio-demographic predictors and average annual rates of caesarean section in Bangladesh between 2004 and 2014. *PLOS ONE*. **12** (5), e0177579. 10.1371/journal.pone.0177579 (2017). 28493956 10.1371/journal.pone.0177579 PMC5426770

[31] Rai, S. D. et al. Caesarean Section rates in South Asian cities: Can midwifery help stem the rise? **6**, 6 (2019).

[32] Ahmmed, F., Manik, M. M. R. & Hossain, M. J. Caesarian section (CS) delivery in Bangladesh: A nationally representative cross-sectional study. *PLOS ONE*. **16** (7), e0254777. 10.1371/journal.pone.0254777 (2021). 34265013 10.1371/journal.pone.0254777 PMC8282068

[33] Aminu, M. et al. Causes of and factors associated with stillbirth in low- and middle-income countries: a systematic literature review. *BJOG: Int. J. Obstet. Gynecol.* **121** (s4), 141–153. 10.1111/1471-0528.12995 (2014). 10.1111/1471-0528.12995 25236649

[34] Hossain, M. A. et al. Rising Trends of Cesarean Section in Bangladesh: Associated Factors and Long-Term Complications on Health of Mother and Children. *J. Maternal Child. Health*. **7** (5), 532–542. 10.26911/thejmch.2022.07.05.04 (2022).

[35] Begum, T. et al. A qualitative study to explore the attitudes of women and obstetricians towards caesarean delivery in rural Bangladesh. *BMC Pregnancy Childbirth*. **18** (1), 368. 10.1186/s12884-018-1993-9 (2018). 30208874 10.1186/s12884-018-1993-9 PMC6134512

[36] Leone, T., Padmadas, S. S. & Matthews, Z. Community factors affecting rising caesarean section rates in developing countries: An analysis of six countries. *Soc. Sci. Med.* **67** (8), 1236–1246. 10.1016/j.socscimed.2008.06.032 (2008). 18657345 10.1016/j.socscimed.2008.06.032

[37] Afiaz, A., Arusha, A. R., Ananna, N., Kabir, E. & Biswas, R. K. A national assessment of elective cesarean sections in Bangladesh and the need for health literacy and accessibility. *Sci. Rep.* **11** (1), 16854. 10.1038/s41598-021-96337-0 (2021). 34413409 10.1038/s41598-021-96337-0 PMC8376956

[38] Woldegiorgis, M. A., Hiller, J., Mekonnen, W., Meyer, D. & Bhowmik, J. Determinants of antenatal care and skilled birth attendance in sub-Saharan Africa: A multilevel analysis. *Health Serv. Res.* **54** (5), 1110–1118. 10.1111/1475-6773.13163 (2019). 31090931 10.1111/1475-6773.13163 PMC6736910

[39] Pulok, M. H., Sabah, M. N. U., Uddin, J. & Enemark, U. Progress in the utilization of antenatal and delivery care services in Bangladesh: where does the equity gap lie? *BMC Pregnancy Childbirth*. **16** (1), 200. 10.1186/s12884-016-0970-4 (2016). 27473150 10.1186/s12884-016-0970-4 PMC4967314

[40] Md. Murtaja Reza Linkon, K. et al. A Study on Factors Associated with Caesarean Section Delivery at Tangail District of Bangladesh. *JHER* **8** (1), 22. 10.11648/j.jher.20220801.14 (2022).

[41] Hasan, F., Alam, M. M. & Hossain MdG. Associated factors and their individual contributions to caesarean delivery among married women in Bangladesh: analysis of Bangladesh demographic and health survey data. *BMC Pregnancy Childbirth*. **19** (1), 433. 10.1186/s12884-019-2588-9 (2019). 31752772 10.1186/s12884-019-2588-9 PMC6873680

[42] Rahman, M. M. et al. Determinants of caesarean section in Bangladesh: Cross-sectional analysis of Bangladesh Demographic and Health Survey 2014 Data. *PLOS ONE*. **13** (9), e0202879. 10.1371/journal.pone.0202879 (2018). 30208058 10.1371/journal.pone.0202879 PMC6135390

[43] Robson, M. S. Can we reduce the caesarean section rate? Best Practice & Research Clinical. *Obstet. Gynecol.* **15** (1), 179–194. 10.1053/beog.2000.0156 (2001). 10.1053/beog.2000.0156 11359322

