# Supplementary material for: From medical necessity to private-sector dominance: a two-decade evolution of caesarean section determinants in Bangladesh (2004–2022)
Source: Sci Rep. 2026 May 9;16:21320. doi: 10.1038/s41598-026-52229-9 (PMC13346757; doi:10.1038/s41598-026-52229-9)
Supplement: Supplementary file 1 — Supplementary Material 1 [file 41598_2026_52229_MOESM1_ESM.docx]

**Supplementary Figure S1.** Trends in caesarean delivery prevalence by place of residence (urban/rural), Bangladesh DHS 2004–2022.


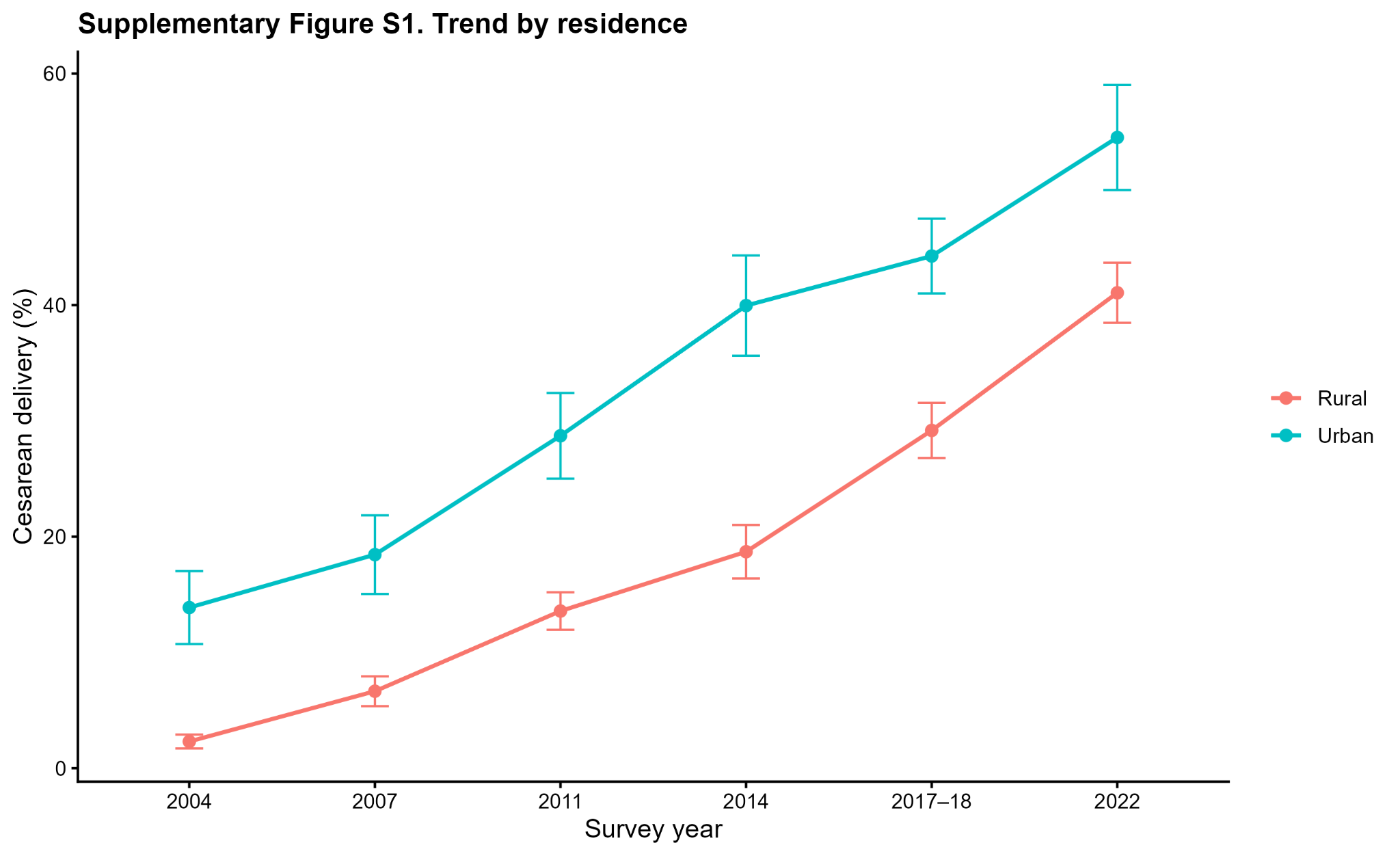


**Supplementary Figure S2.** Trends in caesarean delivery prevalence by place of delivery, Bangladesh DHS 2004–2022.


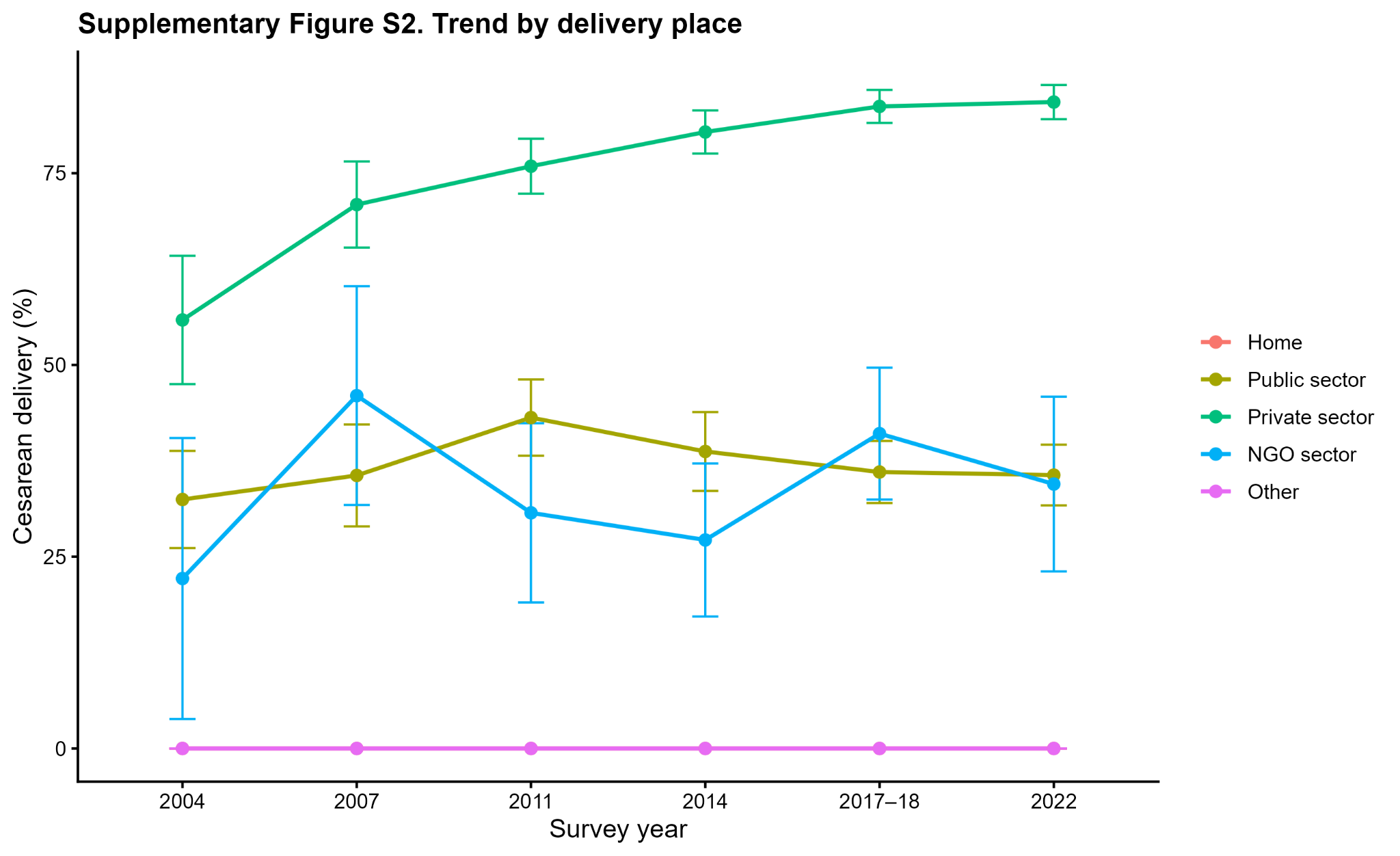


Sensitivity analyses (survey-weighted pooled-period models)

Same covariates as the main model; only the analytic sample changes.

SA1 First births only

| **Characteristic** | **2004–07** | | | **2011–14** | | | **2018–22** | | |
| --- | --- | --- | --- | --- | --- | --- | --- | --- | --- |
|  | **AOR** | **95% CI** | **p** | **AOR** | **95% CI** | **p** | **AOR** | **95% CI** | **p** |
| **ANC Visit** |  |  |  |  |  |  |  |  |  |
| No visit |  |  |  |  |  |  |  |  |  |
| 1–3 visits | 2.29 | 1.04–5.03 | 0.041 | 1.86 | 1.28–2.69 | 0.001 | 2.72 | 1.56–4.74 | <0.001 |
| 4+ visits | 4.71 | 2.10–10.56 | <0.001 | 2.71 | 1.80–4.08 | <0.001 | 3.91 | 2.23–6.86 | <0.001 |
| **BMI** |  |  |  |  |  |  |  |  |  |
| Normal |  |  |  |  |  |  |  |  |  |
| Underweight | 0.78 | 0.53–1.15 | 0.209 | 0.8 | 0.62–1.04 | 0.091 | 0.88 | 0.69–1.12 | 0.312 |
| Overweight | 3.22 | 1.83–5.64 | <0.001 | 1.57 | 1.11–2.21 | 0.011 | 2.19 | 1.69–2.86 | <0.001 |
| Obese | 1.6 | 0.63–4.05 | 0.319 | 2.08 | 1.21–3.55 | 0.008 | 1.5 | 1.23–1.83 | <0.001 |
| **Child Sex** |  |  |  |  |  |  |  |  |  |
| Female |  |  |  |  |  |  |  |  |  |
| Male | 1 | 0.73–1.37 | 0.994 | 1.23 | 1.00–1.50 | 0.045 | 1.11 | 0.94–1.30 | 0.21 |
| **Maternal Age** |  |  |  |  |  |  |  |  |  |
| <20 yrs |  |  |  |  |  |  |  |  |  |
| 20–24 yrs | 1.4 | 0.97–2.04 | 0.075 | 1.53 | 1.21–1.92 | <0.001 | 1.39 | 1.15–1.68 | <0.001 |
| 25–29 yrs | 2.41 | 1.19–4.90 | 0.015 | 2 | 1.35–2.96 | <0.001 | 1.79 | 1.27–2.53 | <0.001 |
| 30–34 yrs | 5.39 | 2.20–13.25 | <0.001 | 11.61 | 3.79–35.56 | <0.001 | 3.92 | 1.53–10.06 | 0.005 |
| 35–39 yrs | 42.65 | 0.04–40518.51 | 0.284 | 13.51 | 3.41–53.46 | <0.001 | 2.63 | 0.48–14.32 | 0.262 |
| 40+ yrs | 0 | 0.00–0.00 | <0.001 | 4268971.94 | 802660.54–22704643.66 | <0.001 | 0 | 0.00–0.00 | <0.001 |
| **Maternal Education** |  |  |  |  |  |  |  |  |  |
| No education |  |  |  |  |  |  |  |  |  |
| Primary | 0.78 | 0.30–2.03 | 0.617 | 1.1 | 0.63–1.90 | 0.747 | 0.89 | 0.45–1.78 | 0.743 |
| Secondary | 1.57 | 0.64–3.84 | 0.327 | 1.22 | 0.71–2.09 | 0.478 | 1.23 | 0.63–2.41 | 0.541 |
| Higher | 2.59 | 0.92–7.32 | 0.074 | 1.5 | 0.82–2.74 | 0.19 | 1.42 | 0.71–2.84 | 0.32 |
| **Media Exposure** |  |  |  |  |  |  |  |  |  |
| No |  |  |  |  |  |  |  |  |  |
| Yes | 0.8 | 0.43–1.49 | 0.482 | 1.31 | 0.99–1.73 | 0.055 | 1.15 | 0.96–1.38 | 0.141 |
| **Partner Education** |  |  |  |  |  |  |  |  |  |
| No education |  |  |  |  |  |  |  |  |  |
| Primary | 1.44 | 0.71–2.90 | 0.309 | 0.78 | 0.54–1.12 | 0.177 | 1.35 | 0.96–1.89 | 0.086 |
| Secondary | 1.44 | 0.72–2.89 | 0.306 | 1.18 | 0.79–1.75 | 0.416 | 1.61 | 1.15–2.25 | 0.005 |
| Higher | 1.47 | 0.69–3.15 | 0.319 | 1.64 | 1.06–2.54 | 0.026 | 2.08 | 1.43–3.03 | <0.001 |
| **Religion** |  |  |  |  |  |  |  |  |  |
| Islam |  |  |  |  |  |  |  |  |  |
| Buddhism | 0 | 0.00–0.00 | <0.001 | 0.64 | 0.20–2.03 | 0.451 | 0.48 | 0.13–1.73 | 0.26 |
| Christianity | 1.25 | 0.21–7.28 | 0.805 | 3.3 | 0.43–25.05 | 0.249 | 1.03 | 0.28–3.74 | 0.965 |
| Hinduism | 1.55 | 0.93–2.58 | 0.092 | 1.47 | 1.02–2.12 | 0.041 | 1.43 | 1.04–1.97 | 0.029 |
| **Region** |  |  |  |  |  |  |  |  |  |
| Central |  |  |  |  |  |  |  |  |  |
| Coastal | 0.4 | 0.26–0.61 | <0.001 | 0.73 | 0.55–0.98 | 0.038 | 0.55 | 0.43–0.72 | <0.001 |
| North | 0.59 | 0.38–0.91 | 0.018 | 0.83 | 0.59–1.16 | 0.271 | 1.12 | 0.86–1.46 | 0.41 |
| South-West/East | 0.64 | 0.40–1.04 | 0.071 | 0.91 | 0.67–1.24 | 0.556 | 1.15 | 0.89–1.50 | 0.287 |
| **Residence** |  |  |  |  |  |  |  |  |  |
| Rural |  |  |  |  |  |  |  |  |  |
| Urban | 1.32 | 0.92–1.89 | 0.132 | 1.07 | 0.83–1.37 | 0.614 | 0.88 | 0.72–1.09 | 0.25 |
| **Wealth** |  |  |  |  |  |  |  |  |  |
| Poorest |  |  |  |  |  |  |  |  |  |
| Middle | 0.98 | 0.34–2.84 | 0.969 | 2.42 | 1.49–3.91 | <0.001 | 1.5 | 1.12–2.00 | 0.007 |
| Poorer | 0.76 | 0.25–2.29 | 0.623 | 2.01 | 1.23–3.29 | 0.005 | 1.36 | 1.02–1.83 | 0.038 |
| Richer | 2.32 | 0.91–5.90 | 0.078 | 2.75 | 1.70–4.44 | <0.001 | 1.61 | 1.17–2.22 | 0.003 |
| Richest | 3.35 | 1.30–8.59 | 0.013 | 4.44 | 2.68–7.37 | <0.001 | 2.24 | 1.58–3.16 | <0.001 |
| **Working Status** |  |  |  |  |  |  |  |  |  |
| No |  |  |  |  |  |  |  |  |  |
| Yes | 0.92 | 0.56–1.52 | 0.743 | 0.92 | 0.66–1.29 | 0.645 | 0.73 | 0.59–0.90 | 0.003 |

SA2 Facility births only (no Home)

| **Characteristic** | **2004–07** | | | **2011–14** | | | **2018–22** | | |
| --- | --- | --- | --- | --- | --- | --- | --- | --- | --- |
|  | **AOR** | **95% CI** | **p** | **AOR** | **95% CI** | **p** | **AOR** | **95% CI** | **p** |
| **ANC Visit** |  |  |  |  |  |  |  |  |  |
| No visit |  |  |  |  |  |  |  |  |  |
| 1–3 visits | 1.46 | 0.77–2.75 | 0.245 | 0.99 | 0.68–1.42 | 0.935 | 1.52 | 0.96–2.42 | 0.074 |
| 4+ visits | 2.08 | 1.08–3.97 | 0.028 | 1.07 | 0.74–1.54 | 0.717 | 1.67 | 1.04–2.66 | 0.032 |
| **Birth Order** |  |  |  |  |  |  |  |  |  |
| 1st |  |  |  |  |  |  |  |  |  |
| 2nd | 0.55 | 0.38–0.79 | 0.001 | 0.63 | 0.50–0.81 | <0.001 | 0.96 | 0.79–1.16 | 0.685 |
| 3rd or higher | 0.3 | 0.18–0.52 | <0.001 | 0.45 | 0.33–0.62 | <0.001 | 0.5 | 0.39–0.65 | <0.001 |
| **BMI** |  |  |  |  |  |  |  |  |  |
| Normal |  |  |  |  |  |  |  |  |  |
| Obese | 1.77 | 0.85–3.68 | 0.127 | 2.16 | 1.45–3.21 | <0.001 | 1.26 | 1.07–1.48 | 0.006 |
| Overweight | 1.45 | 0.95–2.22 | 0.085 | 1.38 | 1.08–1.77 | 0.01 | 1.33 | 1.08–1.65 | 0.008 |
| Underweight | 0.73 | 0.50–1.07 | 0.108 | 0.91 | 0.69–1.20 | 0.52 | 0.81 | 0.62–1.05 | 0.104 |
| **Child Sex** |  |  |  |  |  |  |  |  |  |
| Female |  |  |  |  |  |  |  |  |  |
| Male | 1 | 0.76–1.32 | 0.987 | 1.21 | 1.02–1.43 | 0.026 | 1.09 | 0.96–1.25 | 0.19 |
| **Maternal Age** |  |  |  |  |  |  |  |  |  |
| <20 yrs |  |  |  |  |  |  |  |  |  |
| 20–24 yrs | 1.63 | 1.13–2.37 | 0.01 | 1.24 | 0.96–1.59 | 0.099 | 1.23 | 1.01–1.50 | 0.036 |
| 25–29 yrs | 2.15 | 1.28–3.61 | 0.004 | 2.01 | 1.42–2.85 | <0.001 | 1.4 | 1.10–1.80 | 0.007 |
| 30–34 yrs | 4.29 | 2.23–8.26 | <0.001 | 2.64 | 1.74–3.99 | <0.001 | 1.98 | 1.46–2.67 | <0.001 |
| 35–39 yrs | 2.66 | 1.03–6.88 | 0.044 | 1.96 | 1.04–3.72 | 0.039 | 1.99 | 1.24–3.17 | 0.004 |
| 40+ yrs | 1.9 | 0.41–8.91 | 0.416 | 1.2 | 0.39–3.72 | 0.752 | 1.64 | 0.63–4.23 | 0.311 |
| **Maternal Education** |  |  |  |  |  |  |  |  |  |
| No education |  |  |  |  |  |  |  |  |  |
| Higher | 0.55 | 0.25–1.22 | 0.143 | 1.61 | 1.01–2.58 | 0.048 | 1.17 | 0.77–1.79 | 0.464 |
| Primary | 0.44 | 0.22–0.86 | 0.017 | 1.1 | 0.75–1.61 | 0.639 | 0.93 | 0.63–1.39 | 0.739 |
| Secondary | 0.56 | 0.29–1.09 | 0.089 | 1.47 | 1.00–2.17 | 0.051 | 1.08 | 0.74–1.60 | 0.68 |
| **Media Exposure** |  |  |  |  |  |  |  |  |  |
| No |  |  |  |  |  |  |  |  |  |
| Yes | 0.87 | 0.52–1.43 | 0.577 | 1.02 | 0.79–1.30 | 0.889 | 1.11 | 0.93–1.31 | 0.242 |
| **Multiple Birth** |  |  |  |  |  |  |  |  |  |
| No |  |  |  |  |  |  |  |  |  |
| Yes | 9.58 | 2.51–36.57 | 0.001 | 2.36 | 1.13–4.95 | 0.023 | 1.41 | 0.78–2.57 | 0.26 |
| **Partner Education** |  |  |  |  |  |  |  |  |  |
| No education |  |  |  |  |  |  |  |  |  |
| Higher | 1.93 | 1.03–3.61 | 0.041 | 2.04 | 1.36–3.08 | <0.001 | 1.45 | 1.08–1.95 | 0.015 |
| Primary | 1.71 | 0.96–3.06 | 0.069 | 1.28 | 0.89–1.84 | 0.178 | 1.09 | 0.86–1.39 | 0.471 |
| Secondary | 1.91 | 1.12–3.28 | 0.019 | 1.5 | 1.06–2.11 | 0.022 | 1.07 | 0.84–1.38 | 0.575 |
| **Religion** |  |  |  |  |  |  |  |  |  |
| Islam |  |  |  |  |  |  |  |  |  |
| Buddhism |  |  |  | 0.86 | 0.37–1.98 | 0.718 | 0.26 | 0.09–0.78 | 0.017 |
| Christianity | 1.12 | 0.21–5.85 | 0.895 | 1.26 | 0.38–4.18 | 0.707 | 0.95 | 0.33–2.75 | 0.929 |
| Hinduism | 0.75 | 0.47–1.19 | 0.225 | 0.85 | 0.61–1.18 | 0.334 | 1 | 0.77–1.30 | 0.99 |
| **Region** |  |  |  |  |  |  |  |  |  |
| Central |  |  |  |  |  |  |  |  |  |
| Coastal | 0.53 | 0.36–0.78 | 0.002 | 0.48 | 0.37–0.62 | <0.001 | 0.47 | 0.37–0.59 | <0.001 |
| North | 0.7 | 0.48–1.01 | 0.059 | 0.5 | 0.38–0.65 | <0.001 | 0.98 | 0.78–1.23 | 0.867 |
| South-West/East | 0.51 | 0.33–0.79 | 0.003 | 0.59 | 0.46–0.77 | <0.001 | 0.92 | 0.75–1.13 | 0.44 |
| **Residence** |  |  |  |  |  |  |  |  |  |
| Rural |  |  |  |  |  |  |  |  |  |
| Urban | 0.83 | 0.59–1.15 | 0.26 | 0.72 | 0.58–0.89 | 0.002 | 0.84 | 0.70–1.01 | 0.065 |
| **Wealth** |  |  |  |  |  |  |  |  |  |
| Poorest |  |  |  |  |  |  |  |  |  |
| Middle | 1 | 0.46–2.18 | 0.99 | 1.56 | 1.02–2.40 | 0.041 | 1.46 | 1.15–1.85 | 0.002 |
| Poorer | 0.7 | 0.28–1.77 | 0.452 | 1.33 | 0.80–2.20 | 0.266 | 1.44 | 1.14–1.83 | 0.003 |
| Richer | 0.88 | 0.41–1.88 | 0.742 | 1.52 | 0.97–2.36 | 0.065 | 1.48 | 1.14–1.91 | 0.003 |
| Richest | 1.35 | 0.61–2.96 | 0.457 | 1.87 | 1.14–3.06 | 0.013 | 1.95 | 1.46–2.60 | <0.001 |
| **Working Status** |  |  |  |  |  |  |  |  |  |
| No |  |  |  |  |  |  |  |  |  |
| Yes | 0.89 | 0.57–1.38 | 0.598 | 0.88 | 0.66–1.15 | 0.346 | 0.76 | 0.65–0.89 | <0.001 |
